# Cell division cycle fluctuation of Pal concentration in Escherichia coli

**DOI:** 10.1099/acmi.0.000759.v3

**Published:** 2024-11-13

**Authors:** Laureen M.Y. Mertens, Xinwei Liu, Jolanda Verheul, Alexander J.F. Egan, Waldemar Vollmer, Tanneke den Blaauwen

**Affiliations:** 1 Bacterial Cell Biology and Physiology, Swammerdam Institute for Life Science, University of Amsterdam, Amsterdam, Netherlands; 2 Centre for Bacterial Cell Biology, Biosciences Institute, Newcastle University, Newcastle upon Tyne, UK; 3 Institute for Molecular Bioscience, The University of Queensland, Brisbane, QLD, Australia

**Keywords:** cell division, immunolabelling, outer membrane, periplasm, protease, Tol-Pal

## Abstract

The Tol-Pal proteins stabilize the outer membrane during cell division in many Gram-negative bacteria, including *Escherichia coli*. Pal is an outer membrane lipoprotein that can bind peptidoglycan. It accumulates at the septum during division by a mobilization-and-capture mechanism. This work further substantiates and extends knowledge of Pal’s localization in *E. coli* using immunolabelling; this method enables the detection of endogenous proteins. The midcell localization of Pal and TolB, as seen with fluorescent protein fusions, during cell division, was confirmed. The retention of Pal in newly formed cell poles seemed to persist longer than observed with fluorescent Pal fusions. The concentration of endogenous Pal during the cell division cycle fluctuated: it decreased initially (to half the fluorescence concentration (32.1 au µm^−3^) of the maximum (64.1 au µm^−3^) reached during the cell cycle) and then increased during the second half of the cell division cycle. We probed for possible regulators and proposed two new putative regulators of Pal. By deleting the periplasmic protease, Prc decreased the total Pal abundance (to ~65% of the fluorescence concentration in WT cells) and affected its concentration fluctuation during the cell cycle. This suggests that Prc controls a cell division stage-specific regulator of Pal. Immunolabelling also supported the prediction that the small RNA MicA suppresses Pal expression (the fluorescence concentration of Pal in cells without MicA is double that of Pal in WT cells). However, the regulation by MicA occurred in a cell cycle-independent manner. All these findings urge further research on the tight regulation of the dividing cell envelope stability.

**Impact Statement**

The Gram-negative cell envelope consists of three layers: the inner membrane, the periplasm, containing a peptidoglycan layer, and the outer membrane. This envelope renders Gram-negative bacteria naturally resistant to environmental challenges, including certain antibiotics, and requires tight regulation for cell growth and division. The Tol-Pal machinery stabilizes the outer membrane during and just after cell division by attaching it specifically to the septal peptidoglycan. This study contributes to the understanding of how Pal is regulated in subcellular location, time and abundance. The native Tol-Pal proteins were visualized in cells by immunolabelling. The resulting data substantiated and expanded complementary data generated so far with fluorescent protein fusions. Confirmed was that (i) Pal and TolB accumulate at the septum during cell division, (ii) Pal needs active septum synthesis for its midcell localization and (iii) Pal remains accumulated at the new pole after cell division. New findings are (i) the amount of endogenous Pal changes during the cell cycle, (ii) this change is possibly regulated by the periplasmic protease Prc and (iii) the amount of Pal is downregulated by the small RNA MicA, but this is cell cycle-independent.

## Data Summary

Microscopy images were analysed with ImageJ and the plugin ObjectJ. ImageJ can be downloaded here: https://imagej.net/ij/download.html.

The ImageJ plugin (ObjectJ, Coli-Inspector) used for the analysis of microscopy images can be downloaded here: https://sils.fnwi.uva.nl/bcb/objectj/.

A direct link to the manual of ObjectJ and Coli-Inspector can be read here: https://sils.fnwi.uva.nl/bcb/objectj/examples/Coli-Inspector/Coli-Inspector-MD/coli-inspector.html.

Microscopy images, ObjectJ project files and analysed data are available through Figshare, organized in folders per figure: https://doi.org/10.21942/uva.24802704 [1].].

The authors confirm all supporting data, code and protocols have been provided within the article or through supplementary data files.

## Introduction

The cell envelope of Gram-negative bacteria consists of the inner membrane (IM) and the periplasm with a peptidoglycan (PG) layer attached to the outer membrane (OM). The synthesis and separation of all cell envelope layers at the division site have to be tightly coordinated to enable the growth and survival of the cell in each round of division. We begin to understand how OM biogenesis is coordinated with PG growth [2,4], but how the cell synchronizes these processes during cell division is not fully understood [5]. The Tol-Pal protein machinery is responsible for the transient tethering of the OM to the PG at the septum during cell division [6] and is involved in OM homeostasis and the regulation of PG-remodelling enzymes [7,10].

**Fig. 1.**
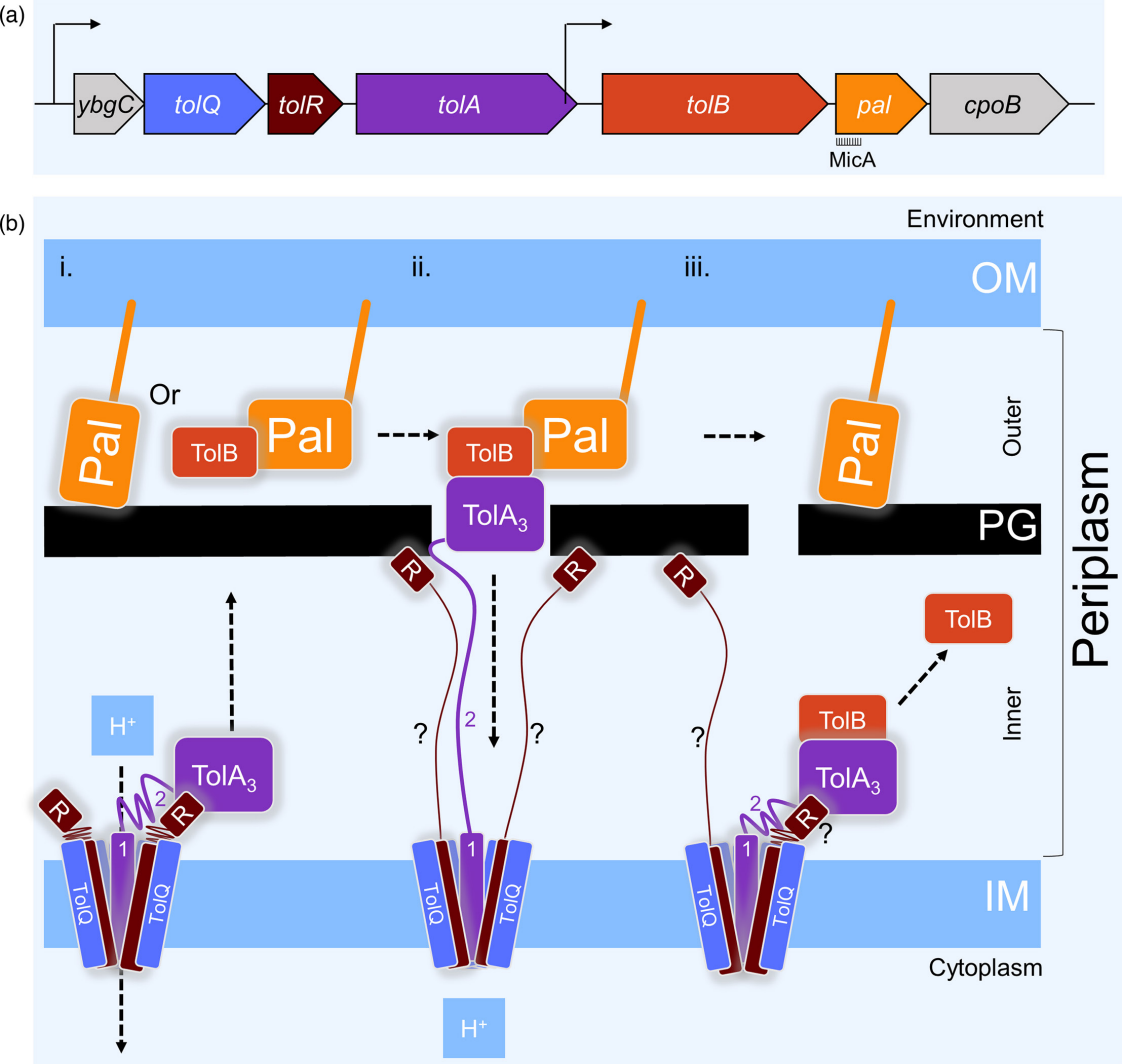
Tol-Pal machinery in *E. coli*. (**a**) Structure of the *tol-pal* operons in the MG1655 *E. coli* genome (NCBI (National Center for Biotechnology Information) GenBank: U00096.3). The Tol-Pal genes are distributed over two adjacent operons: *ybgC-tolQ-tolR-tolA* and *tolB-pal-cpoB*. The small RNA MicA binds in the 5′ UTR of *pal* on the mRNA (MicA is not to scale in the figure). (**b**) Sketch of the mobilization-and-capture mechanism that ensures Pal stabilizes the OM by binding the PG at the septum in the Gram-negative cell envelope. Pal is anchored in the OM with a lipid tail and is either bound to TolB, enabling Pal to diffuse freely in the OM or bound to the PG layer in the periplasm, which strongly limits the diffusion freedom of Pal (**i**). Pal’s affinity for TolB is larger than for PG, but there are far more Pal than TolB proteins in the cell (Table S1). The IM stator TolAQ_5_R_2_ (ratio based on homology to Ton and Mot systems) can engage the proton motive force to extend domain 2 of TolA, so domain 3 (TolA_3_) can reach across the PG layer and detach TolB from Pal (ii). Pal will immediately bind to PG at the site where its TolB gets captured by TolA (iii). Because TolAQ_5_R_2_ accumulates at the site of the division, the capturing of mobile Pal-TolB hetero-dimers and subsequent accumulation of PG-bound static Pal occurs specifically at the septum (ii). Once TolA with the bound TolB has been retracted to beneath the PG, it will release TolB (iii). TolB will diffuse laterally away from the septum (iii) before it can diffuse across the PG, where it will mobilize another Pal protein (**i**), and diffuse until it gets captured at the division site again (ii), depositing another Pal protein to bind the septal PG (iii). TolR likely also undergoes structural changes under the influence of the PMF on the IM stator, and it can bind PG. The nature and function of TolR’s structural changes and binding to PG are unknown and therefore accompanied by a ‘?’ in the figure. Outer, ‘outer periplasm’; Inner, ‘inner periplasm’; H^+^, proton; R, TolR; 1, TolA domain TolA_1_; 2, TolA domain TolA_2_.

The current model for how the Tol-Pal proteins transiently tether the OM to the PG at the division site in *Escherichia coli* is the ‘mobilization-and-capture mechanism’ [11]. The proteins involved are TolA, TolQ and TolR (which reside in the IM), TolB (fully periplasmic) and Pal (OM-anchored) (Fig. 1). TolQ, TolR and TolA are predicted to organize into a proton motive force (PMF)-powered stator complex (Fig. 1) [12]. A proton flux results in structural changes in TolA and TolR that allow TolA to extend across the PG layer, where it binds the periplasmic protein TolB, releases it from the OM-anchored Pal and pulls TolB beneath the PG. The release of TolB from Pal allows Pal to bind the PG – as these interactions are mutually exclusive [13,14]. PG-bound Pal is far less mobile than when bound to TolB [11,15]. During the pre-divisional stages, TolQ, TolR and TolA accumulate at midcell and displace TolB from Pal, particularly at the septum. Pal can then bind to PG [11], tethering the OM to the septal PG during cell division. The PG at midcell will be remodelled into a septum and eventually into two new poles. The increased attachment of the OM to the PG by accumulated Pal at the forming septum is thought to stabilize the OM during all these remodelling steps [11,12]. After its detachment from Pal by TolA, TolB is released from TolA beneath the PG. It is estimated that ~50 kDa globular proteins [16] can pass through the largest holes in the PG network, but the search for holes in the network of suitable size delays diffusion across the periplasm. This delay ensures that TolB (46 kDa), after being released from TolA beneath the PG, first laterally moves away from the septum before diffusing across the PG, where it can bind and mobilize a PG-bound Pal molecule [11].

Pal is an OM-anchored lipoprotein that can non-covalently bind both TolB [17,18] and the mDap residues in PG peptide side chains [14], but these interactions are mutually exclusive. Pal’s affinity for TolB is predicted to be significantly higher than for PG (Kd ~30–38 nM [11,13] versus an estimated 2.2 µM [19], respectively). Because the amount of Pal molecules is predicted to be seven to ten times in excess of TolB (Table S1, available in the online Supplementary Material [20]), a large portion of Pal will remain unbound by TolB and be bound to PG. The deletion of *pal* results in the same phenotypes known for the deletion of single or all the *tolABQR* genes: increased OM permeability and sensitivity to detergents [21,22], shedding of OM vesicles [22,23] and cell separation defects [24,25]. Pal is not the only anchorage of the OM to the PG. Lpp and OmpA are also abundant OM proteins that can bind PG. The observation that overexpression of Pal can rescue an *lpp* null mutation, whilst the overexpression of Lpp cannot rescue a *pal* null mutation, resulted in the hypothesis that Pal has an additional function to Lpp [22]. A role of the Tol-Pal genes in OM stabilization during cell division was predicted [24]. The nature of this involvement in cell division has been substantiated in recent years [9,11,13, 15, 26], resulting in the model described earlier: the mobilization-and-capture mechanism (Fig. 1) [6,11].

Functional fluorescent fusions to all components of the Tol-Pal machinery (TolA, TolB, TolQ, TolR and Pal) expressed from either plasmids or their genomic locus revealed that they all accumulate at the septum during cell division [11,15, 24, 26,28]. In contrast to the Tol components, Pal was retained at the new cell pole for up to 20% of the cell cycle [26]. Time-lapse microscopy during two generations of a single cell from the same population suggested that the decrease in polar localization correlated with midcell accumulation [26].

Fluorescent fusions to TolA and TolQ were the only two Tol-Pal proteins that could still localize at midcell without one or all of the other proteins of the Tol-Pal machinery [26,28]. TolR needs TolA or TolQ, while Pal requires all four Tol proteins to accumulate at the division site [24,26]. The interaction of Pal with PG (Pal amino acid residue R104) is not required for its recruitment to the division site. However, its interaction with TolB (E102) and consequential Pal mobilization is needed for Pal to accumulate to the forming septum [26]. The interaction of Pal with PG (through R104) is required to prevent OM blebbing at the septum, confirming that Pal is responsible for OM stabilization to the septal PG during cell division [26]. Pal has two states of mobility: when it is attached to PG, it diffuses very slowly through the OM; in its other mobile state – likely bound to TolB – it diffuses much faster [11]. It was particularly fast away from the septum (8×10^−4^ µm s^−1^) and slower close to the septum (4×10^−4^ µm s^−1^) in dividing cells. In non-dividing cells, Pal migrates at an even slower but constant speed along the entire cell axis (2×10^−4^ µm s^−1^) [11].

TolA and TolQ require the periplasmic peptide of FtsN (^E^FtsN), essential for cell division, to localize at the septum [24]. FtsN is the last essential component of the divisome to appear at the cell division site. The primary function of FtsN is the activation of dominant divisome PG synthetases FtsI and FtsW that accumulate at midcell before FtsN. TolA and TolQ are most likely attracted to the septum by the active synthesis of septal PG, not by direct protein–protein interactions with FtsN [24,28]. The inhibition of the prime septal transpeptidase FtsI (penicillin-binding protein 3 (PBP3)) activity with aztreonam stopped TolA and TolQ from sequestering at midcell [28]. Additionally, it was confirmed that TolA and TolQ can still accumulate at the septum in the absence of FtsN [28]. Therefore, it is not FtsN itself that attracts TolA and TolQ to the septum, but rather the active synthesis of septal PG.

Until now, only fluorescent protein fusions have been used to localize the Tol-Pal proteins [11,15, 24, 26,28, 30]. Our work further substantiates established knowledge of Pal’s midcell localization during cell division, using a complementary technique: immunolabelling. The retention of Pal in newly formed cell poles seemed to persist longer than observed with fluorescent Pal fusions. Immunolabelling also revealed that the amount of Pal per cell volume first decreases and then increases during the cell division cycle. We probed for possible regulators of this fluctuation. Gene deletion of the periplasmic protease Prc decreased the Pal abundance detected with immunolabelling, and the changes in Pal concentration during the cell cycle were less pronounced. This implies that Prc controls a regulator of Pal. We also confirmed that the small RNA (sRNA) MicA does indeed decrease the amount of Pal found with immunolabelling, but not in a cell cycle-dependent manner. As expected [28], Pal localization was lost when cells were treated with aztreonam. Aztreonam treatment also resulted in increased amounts of Pal detected with immunolabelling.

## Methods

### Strains and growth conditions

All *E. coli* strains used in this study are listed in Table 1. LMC500 was used as a WT for the study of the spatiotemporal behaviour of Pal. All deletion mutants were derived from BW25113, in which Pal behaved similarly as in LMC500. It was therefore not considered necessary to transduce these gene deletions to an LMC500 background. *ΔmicA* was constructed according to Datsenko and Wanner [31]. Primers GD-587 (TTTTTTAAAAATTTTCTGAACTCTTTCTTCCCAGGCGAGTCTGAGTATATgcgattgtgtaggctggagc) and GD-588 (GATACCGAACCGTTTGCGGTGTGGCTGGAAAAACACGCCTGACAG-AAAAGcatggtccatatgaatatcctcc) were used to PCR amplify the kanamycin resistance cassette, flanked by FRT-sites from pKD4 (capitalized bases match up- and downstream sequences of *micA*, lowercase bases to pKD4). The resulting PCR product was transformed in BW25113 carrying the pKD46 plasmid to express the λ-Red genes. The success of the chromosomal deletion of *micA* was confirmed with primers GD-588 (see before) and GD-589 (TTAGCCACCTCCGGTAATTTTT). NT10016 (Δ*prc*) was constructed as described in [32] by transducing BW25113 with P1 lysates [33] of the Δ*prc::cat* strain in a BW38029 background [34].

**Table 1.** Strains used in this study

Strain	Genotype	Reference
BW25113	F-, Δ(*araD-araB)567,* Δ*lacZ4787(::rrnB-3), λ-, rph-1,* Δ(*rhaD-rhaB)568, hsdR514*	[31]
LMC500	MC4100, F-, *araD139,* Δ(*argF-lac)U169, deoC1, flbB5301, lysA1, ptsF25, rbsR, relA1, rpsL150*	[51]
Δ*pal*	BW25113 Δ*pal-790::kan*	[37]
Δ*tolA*	BW25113 Δ*tolA-788::kan*	[37]
Δ*tolB*	BW25113 Δ*tolB-789::kan*	[37]
Δ*nlpI*	BW25113 Δ*nlpI::tet*	[32]
Δ*prc*	BW25113 ∆*prc::cat*	This work
Δ*cpoB*	BW25113 Δ*ybgF::kan*	[40]
Δ*zapA*	LMC500 Δ*zapA::cat*	[52]
Δ*micA*	BW25113 Δ*micA::kan*	This work

*E. coli* K12 cells were grown to a steady state at 28 °C under constant agitation [35] in glucose minimal medium (GB-1) containing 4 g glucose (Roth), 4.83 g K_2_HPO_4_ (VWR), 2.95 g KH_2_PO_4_ (Fisher Chemical), 1.05 g (NH_4_)_2_SO_4_ (Sigma-Aldrich), 0.10 g MgSO_4_·7H_2_O (Roth), 0.28 mg FeSO_4_·7H_2_O (Sigma-Aldrich), 7.1 mg Ca(NO_3_)_2_·4H_2_O (Sigma-Aldrich), 4 mg thiamine (Sigma) and 50 mg lysine (Sigma) per litre. For BW25113 and derived strains, GB-1 was supplemented with 2 µg uracil (Sigma-Aldrich), 20 µg thymidine (Sigma-Aldrich), 50 µg l-arginine (Sigma-Aldrich) and 50 µg l-glutamine (Sigma-Aldrich) per litre water. Growth was followed with OD measurements at 450 nm. For precultures and the Δ*pal* samples used for anti-Pal purification, cells were grown in TY medium (half-salt LB), which contains per litre: 5 g yeast extract (Fisher Bioreagents), 10 g tryptone (Duchefa) and 5 g NaCl (Acros Organics), pH 7.0; the OD of TY cultures was measured at 600 nm. When strains contained chromosomal antibiotic markers, the antibiotic was supplied in the medium in appropriate concentration: 2 µg ml^−1^ tetracycline (Sigma-Aldrich), 5 µg ml^−1^ kanamycin (Sigma) and 5 µg ml^−1^ chloramphenicol (Sigma).

### Immunolabelling

The procedure for immunolabelling used here was previously published by Buddelmeijer *et al.* [36] but will be briefly described here. After reaching a steady state, cells were fixed for 15 min by the addition of a mixture of formaldehyde (final concentration: 2.8%) (Sigma-Aldrich) and glutaraldehyde (final concentration: 0.04%) (Merck) to the shaking culture in the water bath. Excess formaldehyde and glutaraldehyde were removed by three washes with PBS [140 mM NaCl (Acros Organics), 2.7 mM KCl (VWR), 10 mM Na_2_HPO_4_·2H_2_O (Merck), 2 mM KH_2_PO_4_ (Fisher Chemical); pH=7.2]. The cell envelope was then stepwise permeabilized with Triton-X100 (Sigma-Aldrich) (for membrane permeabilization) and lysozyme (Sigma-Aldrich) (for permeabilizing the PG layer). Immunolabelling with rabbit polyclonal antibodies specific against Pal (Fig. S1), FtsZ, FtsN, TolB (Fig. S2) or ZapA was done in blocking buffer [0.5% (w/V) blocking reagent (Boehringer) in PBS; see Table 2 for appropriate dilution rates]. Unbound antibodies were washed away with PBS. To allow for fluorescence microscopy, a secondary antibody, donkey anti-rabbit conjugated to Cy3 [Cy3 AffiniPure Donkey Anti-Rabbit IgG (H+L), Jackson Immunochemistry] in the same blocking buffer was used. After removing excess antibodies by washing them with PBS, cells could be imaged with a fluorescence microscope.

**Table 2.** Antibodies used in this study

Antibody	Monoclonal/polyclonal and host species	Used dilution for immunolabelling	Reference/manufacturer
αPal	Polyclonal, rabbit	1 : 500	[2]
αZapA		1 : 500	[52]
αFtsN		1 : 500	[45]
αFtsZ		1 : 500	[53]
αTolB		1 : 200	[40]
Cy3 AffiniPure Donkey Anti-Rabbit IgG (H+L)	Polyclonal, donkey	1 : 300	Jackson, ImmunoResearch, Europe Ltd, AB_2307443

### Microscopy

For immunolocalization and imaging of Bodipy-C12 staining, labelled cells were trapped between a cover slip and a slab of 1% agarose in PBS on a microscopy slide and photographed with an Orca Flash 4.0 (Hamamatsu) CCD camera mounted on an Olympus BX-60 fluorescence microscope through a 100 ×/N.A. 1.35 oil objective. For fluorescence imaging of Cy3 (immunolabelling) and Bodipy, a red filter cube was used with the following properties: excitation (ex), 560±40 nm; dichroic mirror (dm), 585 nm and emission (em), 630±75 nm). Images were taken using the program ImageJ (http://imagej.nih.gov/ij/) (version 1.53e) with MicroManager (https://www.micro-manager.org).

### Image analysis

Hyperstacks of microscopy images comprising a phase contrast and a fluorescence channel were compiled with ImageJ. Images were scaled to 15.28 µm/pixel. The fluorescence background signal was subtracted with modal values from the fluorescence images as described in [35], and small misalignments of the phase contrast and fluorescence channel were corrected using fast-Fourier techniques as described [35]. The images were then analysed with Coli-Inspector running in combination with the plugin ObjectJ (version 03 and 04; https://sils.fnwi.uva.nl/bcb/objectj/) [35].

The progression of the cell cycle is given as a percentage of cell division cycle age, based on cell length [35]. Where 0% is the shortest cells: a cell starting its life cycle, and 100% is the longest cells: the very final stage of cell division, before two daughter cells are fully separated. Cells are ranked according to their length, and their cell division cycle age can be calculated with the following formula [35]:

$$Cell\;division\;cycle\;age=\frac{ln\left(1-\frac{0.5*rank}{nCells-1}\right)}{\ln\left(0.5\right)}*100$$

## Results

### Pal accumulation changes from new cell pole to midcell during the cell cycle

Fluorescent protein fusions to Pal were found to accumulate at midcell during cell division [24] and to be retained at newly formed poles after division, longer than the inner membrane Tol complex proteins TolA, TolR and TolQ [26]. In the current work, Pal localization was visualized with immunolabelling, which allows for studying the behaviour of native Pal. Immunolabelling comes with the concession that cells must be fixed and subsequently permeabilized for epitope accessibility for the relatively bulky antibodies. Still, it can provide complementing evidence for results found with fluorescent fusion-based work [36].

To verify whether the antibodies against Pal (previously used in western blotting [2]) were suitable for immunolabelling, antibodies were adsorbed to a strain lacking the *pal* gene (Δ*pal*) [37]. The antibodies that did not bind to these cells – present in the supernatant – were used for immunolabelling the WT *E. coli* strain BW25113 grown to a steady state in minimal glucose medium (GB1) at 28 °C. Labelling of the Δ*pal* strain with this supernatant, but also with the crude serum, showed only a barely detectible background signal and no specific fluorescence, which confirmed that the antibodies were highly specific for Pal and did not cross-react to other proteins in the cell (Fig. S1). For all further immunolabelling experiments, crude serum was used.

**Fig. 2.**
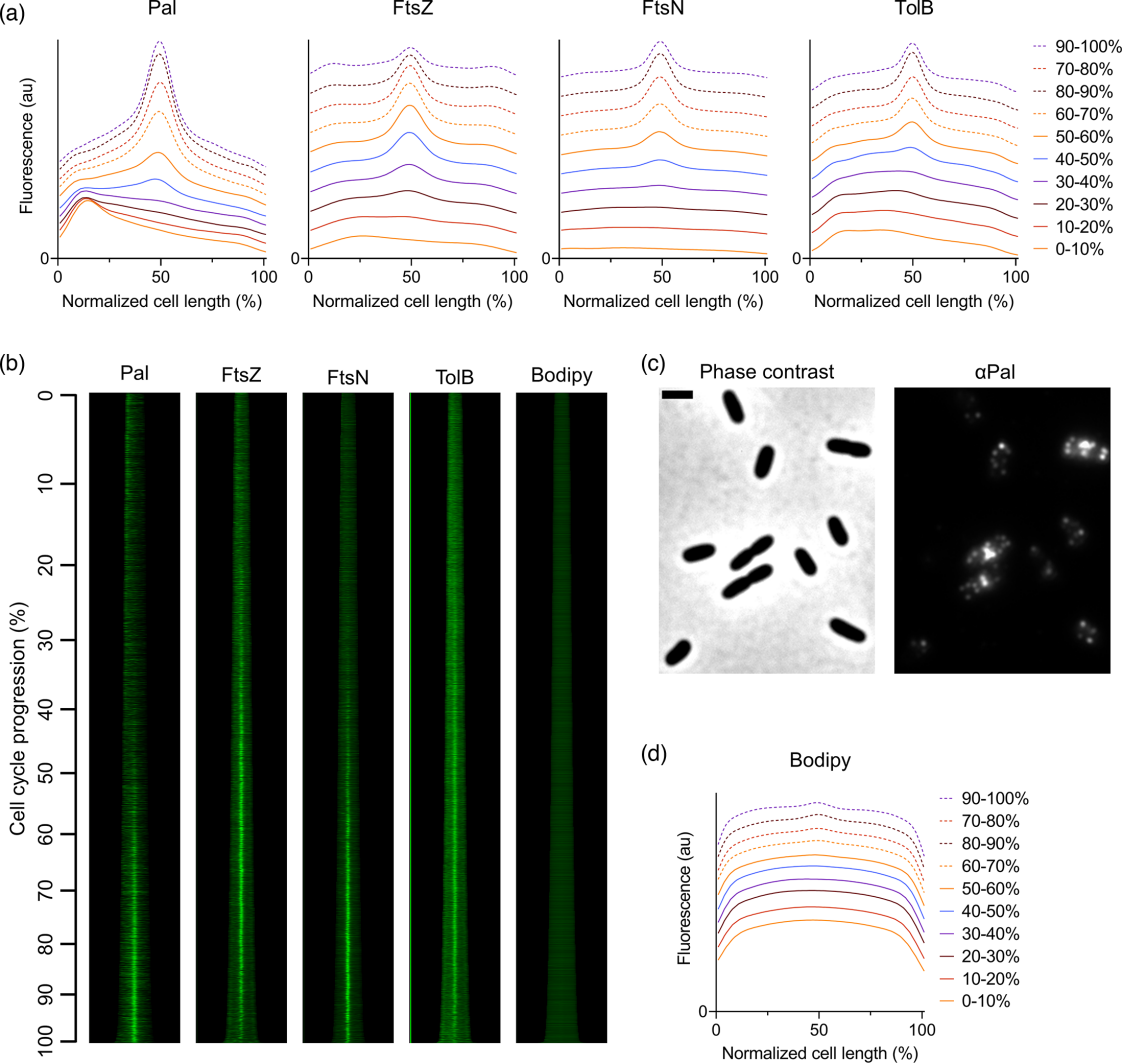
Pal accumulation at midcell coincides with late divisome subunit localization, but it does not delocalize before the completion of cell division; on the contrary, it remains associated with the new cell pole until 40% of the next cell division cycle. LMC500 cells grown to a steady state in minimal glucose medium at 28 °C were immunolabelled with antibodies specific for Pal (*N*=5085 cells) (see Fig. S1 for proof of Pal specificity). For comparison, LMC500 cells labelled with membrane stain Bodipy (*N*=5 382), antibodies specific for FtsZ (*N*=3 300), FtsN (*N*=5 921) and TolB (*N*=5 459) (see Fig. S2 for proof of TolB specificity) were included as well. Fluorescence profiles of cells labelled with antibodies (**a**) specific against Pal, FtsZ, FtsN and TolB (from left to right) and the membrane stain Bodipy (**b**). Cells were binned in 10% age groups, and for each group, an average fluorescence profile was made and plotted against the normalized cell length. To allow better discrimination of the various profiles, each profile has been shifted up with several arbitrary units. (Fig. S3A shows fluorescence profiles without this adjustment). (**b**) Sorted fluorescence demographics corresponding to fluorescence profiles displayed in (**a**) and (**d**). Cells were sorted based on cell length (shortest cells at the top, longest at the bottom; cell cycle progression indicated on the left), and their most fluorescent half is oriented to the left. Corresponding diameter demographics are displayed in Fig. S3B. (**c**) Representative phase contrast and fluorescence images of cells labelled with antibodies specific against Pal. The scale bar equals 2 µm. The data of FtsZ- and FtsN-labelled cells presented in this figure were already part of an earlier publication [44], and the data of Bodipy-stained cells [40] have been inserted here to allow for the direct comparison of Pal localization.

Immunolabelling of Pal confirmed its accumulation at midcell during cell division simultaneously with the late localizing divisome protein FtsN (Fig. 2). The retention of immunolabelled native Pal at newly formed cell poles is more prominent and persistent (Fig. 2a, b) than in kymographs for the Pal-mCherry fusion in earlier work [26]. Pal accumulation at one of the cell poles persists up to ~40% of the cell division cycle (Fig. 2a, b), just before the accumulation of Pal at midcell begins. This resembles what was seen previously in time-lapse microscopy [26], but based on a demographic comprising 5085 cells, one can state this with more confidence than based on time-lapse microscopy of a single cell.

The sudden change in osmolarity sometimes causes freely diffusing periplasmic proteins to accumulate in the cell poles after fixation by formaldehyde or ethanol [38,39]. We observed some Pal localization in the poles of young cells that could either be a remnant of the division site or be due to the fixation procedure. However, this polar localization was never observed for the OM lipoproteins LpoB and NlpI [32,40]. Therefore, we assume that the localization of OM-anchored Pal visualized with immunolabelling reflects its accurate localization.

In conclusion, Pal localizes strongly at one of the poles of cells early in the cell cycle and then accumulates in the middle of cells in the second half of the cell cycle. It is hardly present in the lateral wall during all cell cycle stages.

#### Pal abundance fluctuates during the cell cycle

Abundancies of native proteins during the cell cycle can be determined with immunolabelling, microscopy and subsequent image analysis [35]. Fusions to fluorescent proteins can change both expression levels and the lifespan of proteins in the cell compared with the native protein. Here, we used ObjectJ software to determine the amount of Pal per cell volume (au µm^−3^) of each cell as a function of the cell division cycle age (see ‘Methods’) [41].

**Fig. 3.**
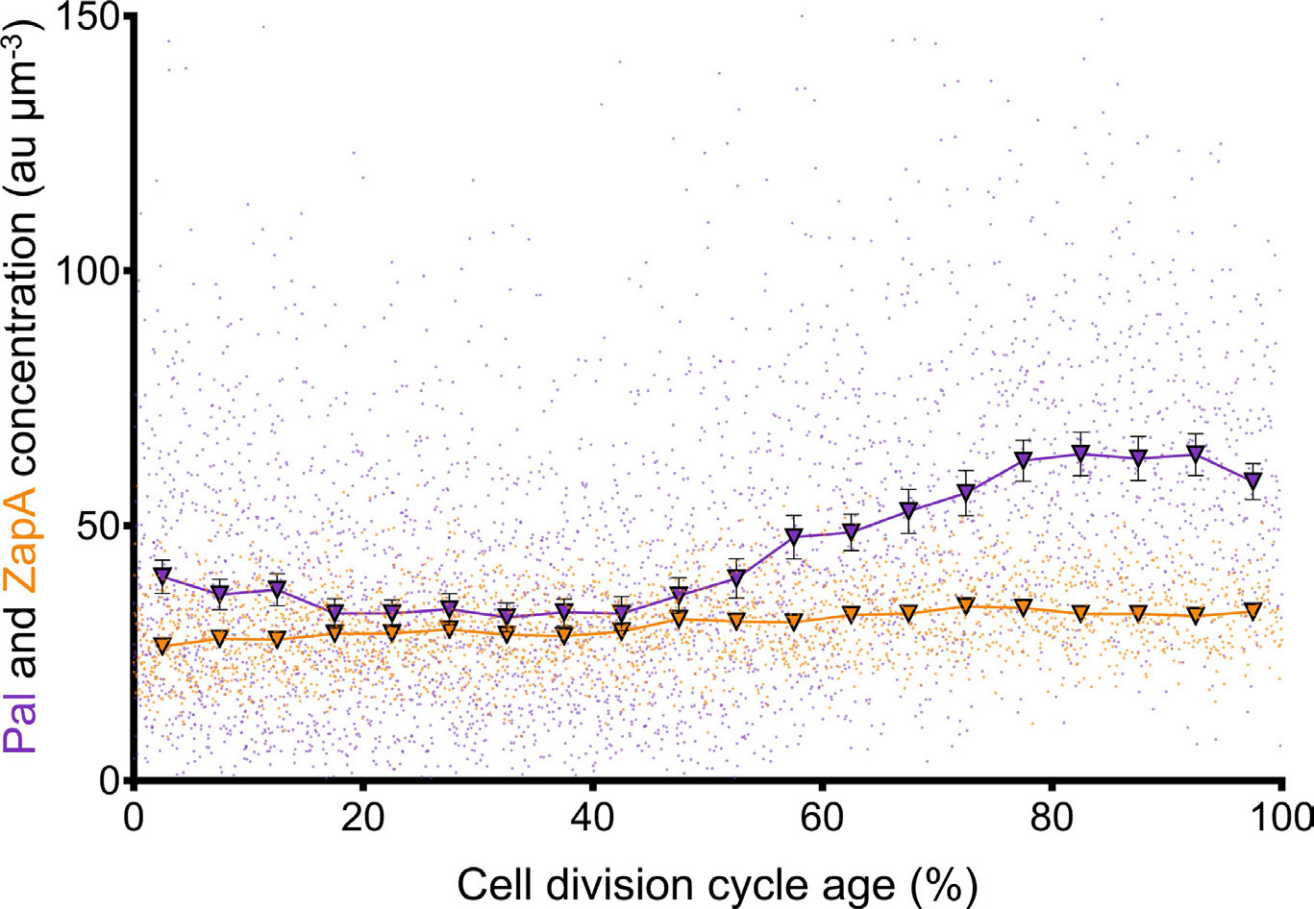
Pal concentration is not constant during the cell division cycle. The fluorescence concentration of Pal (*N*=3681 cells) and ZapA (*N*=2288 cells) is shown in purple and orange, respectively. LMC500 was grown to a steady state in a minimal medium (GB1) at 28 °C and immunolabelled with specific antibodies against the indicated proteins. The unlinked dots are the values measured for the individual cells, and the triangular markers with a black edge connected by a line are the means of 5% age bins with their respective 95% confidence interval error bars. Some error bars are smaller than the marker and therefore not displayed. A 2.7% fraction of cells labelled with anti-ZapA remained unlabelled; therefore, cells with a ZapA concentration <8.0 au µm^−3^ were omitted (Fig. S4).

The concentration of Pal first decreased and later increased as a function of cell division cycle age with a minimum between 25 and 45% and a maximum at 80% of the cell division cycle (Fig. 3). Like FtsZ, PBP3 and PBP5 [35], the synthesis of Pal seems to be upregulated during division. The Pal concentration only decreased at the very end of the cell cycle. As a control, the cell division protein ZapA, which has a stable abundance during the cell cycle, was also immunolabelled in the same experiment as Pal. The analysis of microscopy images showed that its abundance was constant during the cell cycle (Fig. 3). This revokes that the fluctuation in abundance observed for Pal could be attributed to changes in the permeability of the cell envelope for antibodies during the different cell cycle stages.

#### Aztreonam treatment deregulates Pal concentration and localization

Aztreonam is a β-lactam antibiotic [35] that specifically inhibits the transpeptidase activity of the essential protein FtsI (also PBP3) but does not affect its recruitment to a division site [36]. FtsI crosslinks peptide chains of PG at division sites, in complex with FtsW (the essential glycosyltransferase active at the cell division septum). Cells treated with aztreonam can no longer complete cell division and typically elongate, while at least part of the divisome still localizes at midcell and future division sites [36–39].

**Fig. 4.**
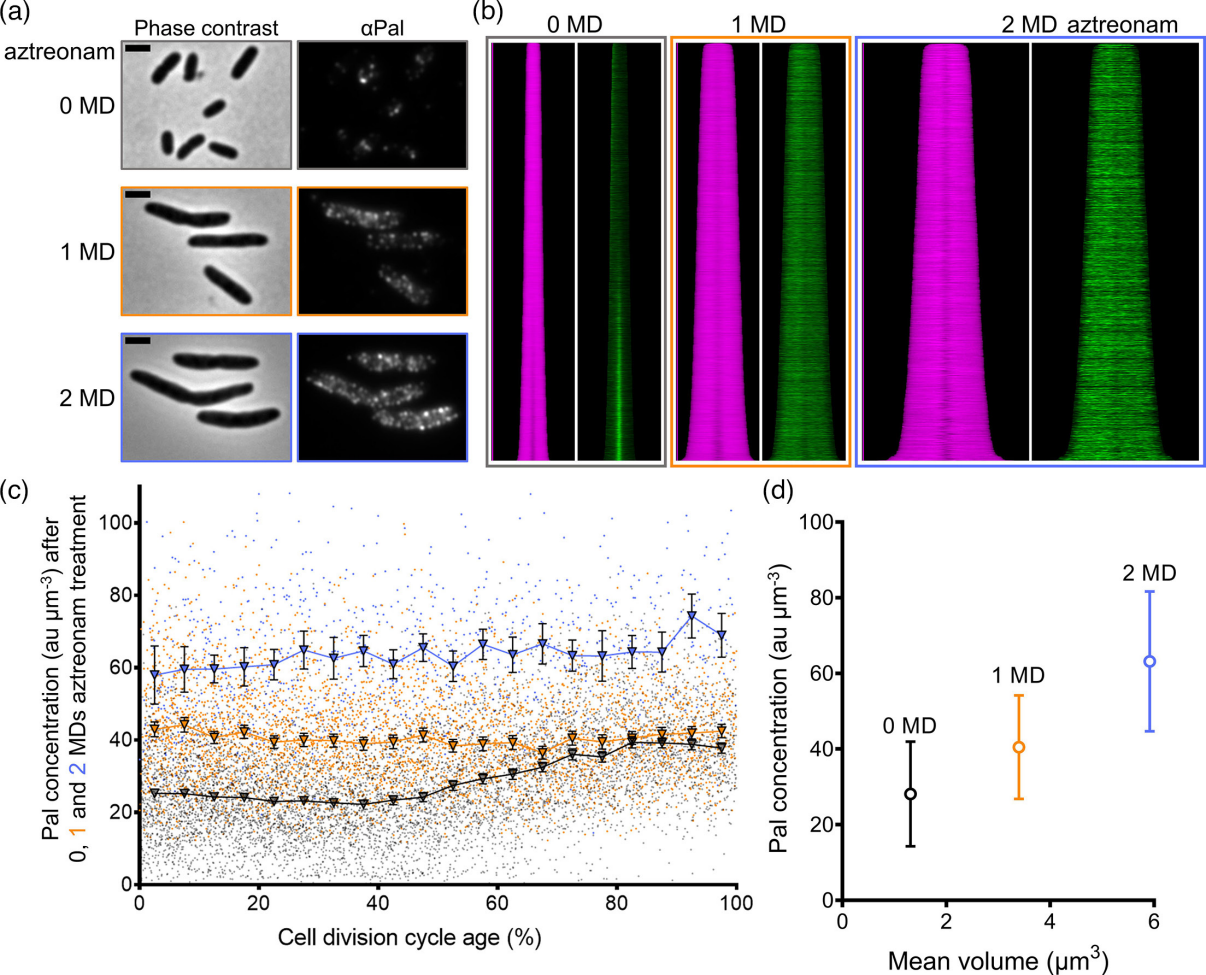
The concentration of Pal is constant during the cell cycle in PBP3-inhibited cells but increases with treatment time. LMC500 was grown to a steady state in a minimal medium (GB1) at 28 °C treated for 0 (grey), 1 (orange) and 2 (blue) mass doublings (MD) with 1 µg ml^−1^ aztreonam that inhibited the cell division-specific class B PBP3, and the samples were immunolabelled with specific antibodies against Pal. (**a**) Representative microscopy images of the samples, phase contrast shown left of the fluorescence microscopy image. (**b**) Diameter profile map (magenta) and fluorescence profile map (green). The profiles or the cells are sorted according to their length, with the most fluorescent half oriented to the left (**c**) Concentration of Pal in arbitrary units per µm^3^ plotted against the normalized cell division cycle age. The colours of the markers correspond to the samples treated with aztreonam for 0 MD (grey), 1 MD (orange) and 2 MD (blue), respectively. Dots represent values measured of individual cells, whilst the triangular markers correspond to the mean Pal concentration for 5% age bins, with their 95% confidence interval displayed as error bars. Some error bars are smaller than the marker and therefore not visible. (**d**) The average concentration of Pal in the cells is plotted against the average volume of the cells; error bars represent the sd. Above the data points, the aztreonam incubation time is indicated. The number of analysed cells for 0, 1 and 2 MD aztreonam treatment is *N*=7677, *N*=3965 and *N*=951, respectively.

Here Pal was immunolabelled in steady-state grown LMC500 cells that were treated with aztreonam for 0, 1 and 2 mass doublings (Fig. 4). Pal no longer accumulates in cell poles in the first half of the cell cycle nor at midcell in the second half (Fig. 4). The changes in concentration seen during the cell cycle (Fig. 3) are also lost in aztreonam-treated cells (Fig. 4). Based on immunolabelling, it seemed that the average concentration of Pal increased ~1.5 times per mass doubling time during the treatment course (Fig. 4c). Pal could be overexpressed in reaction to FtsI inhibition and consequential envelope stress. Another possible explanation for the ongoing increase of Pal concentration in response to aztreonam is that an unknown Pal regulator can no longer degrade Pal – the decrease observed in the first 20% of the cell cycle (Fig. 3) suggests that such a regulator could exist.

#### Prc and MicA dependency of the Pal abundance

The initial decrease and later increase of the Pal concentration to a maximum towards the end of the cell cycle imply that a regulatory mechanism is in place. In this work, we studied the effects of deleting the periplasmic protease Prc (also known as the tail-specific protease), its adapter protein NlpI and the sRNA MicA on Pal concentration in the cell.

The decrease in Pal concentration during the first stages of the cell cycle (Fig. 3) indicated that Pal might be specifically degraded by a periplasmic protease for a short period after cell division. A candidate protease for this function is Prc. Prc is involved in the maturation of divisome protein FtsI; it cleaves off its C-terminus [40] and shows this activity towards other proteins *in vitro*. Prc also regulates the amount of endopeptidase MepS, together with NlpI, which brings Prc and MepS together [41]. NlpI/Prc seems to function as a general regulator of PG hydrolases and synthases involved in cell elongation and division [32,42], and therefore, Prc, by proxy, could be considered a regulator of cell division, and it is therefore the most interesting periplasmic protease to verify. To substantiate whether Prc regulates the abundance of Pal during the cell cycle, Pal was immunolabelled in Δ*prc*, Δ*nlpI* and their parental strain BW25113 (WT). The Δ*prc* and Δ*nlpI* strains contain less Pal than WT at all cell cycle stages (Figs S5 and S6). Δ*nlpI* maintained the decrease and increase of Pal concentration during the cell cycle, whereas this pattern was almost completely lost in Δ*prc*. The Pal concentration did not decline at the beginning of the cell cycle in Δ*prc* and only increased moderately towards the end. Although overall Pal levels were reduced in both deletion strains, the demographics with an enhanced anti-Pal signal show that the accumulation of Pal changed from being present at one of the cell poles to the middle of the cell (Fig. S5a), albeit to a lower concentration in Δ*prc*.

To exclude that Pal levels were changed in Δ*prc* and Δ*nlpI* as a consequence of increased amounts of the endopeptidase MepS, Pal was also immunolabelled in Δ*mepS*. The Pal abundance in Δ*mepS* was lower than in WT but higher than in both Δ*nlpI* and Δ*prc* (Fig. S5), implying that the increased endopeptidase activity of MepS in Δ*nlpI* and Δ*prc* did not exclusively cause the changes seen in both Δ*nlpI* and Δ*prc*. Because the effects of Δ*prc* were the most detrimental of the three deletion strains Δ*mepS*, Δ*nlpI* and Δ*prc*, the follow-up experiments involved only Δ*prc*.

**Fig. 5.**
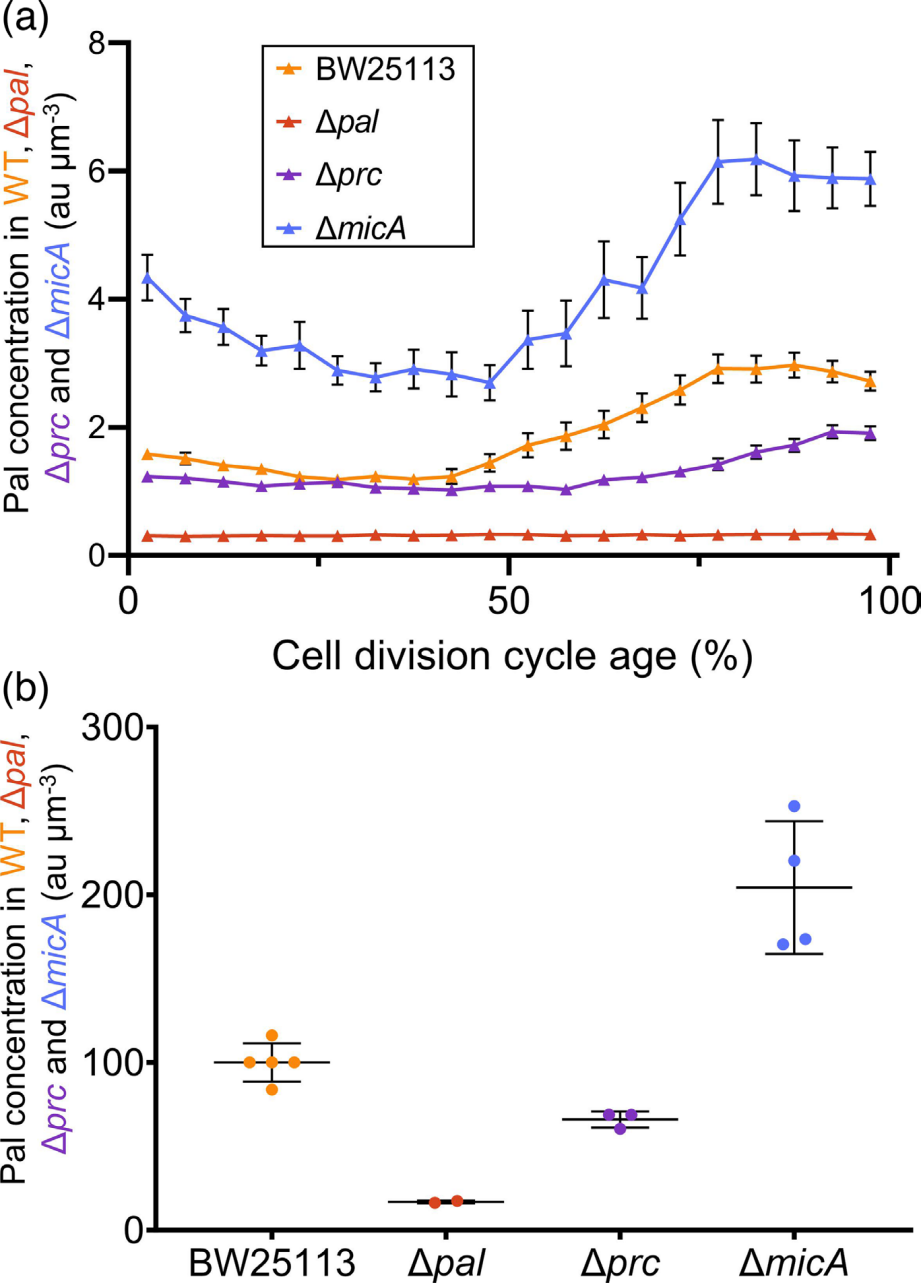
The protease Prc and sRNA MicA regulate the abundance of Pal. (**a**) Concentration of Pal in arbitrary units per µm^3^ plotted against the normalized cell division cycle age. The connected triangular markers correspond to the mean Pal concentration for 5% age bins, with their 95% confidence interval displayed as error bars. Some error bars are smaller than the marker and therefore not visible. The dataset shown in A is ‘set 1’, as described in Table S2. The colours of the markers correspond to BW25113 (bright orange, *N*=3519 cells), Δ*paI* (muted orange, *N*=3941), Δ*prc* (lilac, *N*=6752) and Δ*micA* (blue, *N*=2217). (**b**) Normalized concentrations of Pal in arbitrary units per µm^3^ for BW25113, Δ*paI*, Δ*prc* and Δ*micA* as measured across several experiments. The error bars represent the sd. Table S2 summarizes the datasets included in this graph, and Fig. S7 shows all the individual datasets. Representative microscopy images can be found in Fig. S8.

Another putative regulator of Pal abundance during the cell cycle is the sRNA MicA. MicA was predicted to be complementary to the RBS region of Pal (Fig. 1.) and thereby control the expression of Pal [43]. The interaction of MicA with the RBS of Pal was later experimentally confirmed [38]. To determine whether MicA does indeed downregulate Pal and whether the nature of this regulation changes during the cell cycle, Pal was immunolabelled in a Δ*micA* strain. The overall concentration of Pal was increased during all cell division cycle stages in Δ*micA* compared with its parental BW25113 (Fig. 5a) while maintaining the concentration fluctuation during the cell cycle. The shape of the increase in the plots was slightly altered, but the overall trend remained. The immunolabelling of Pal in Δ*micA* showed that the sRNA regulates Pal expression, but it did not affect the Pal abundance during the cell cycle.

To confirm that the decrease and increase in Pal concentration in, respectively, the Δ*prc* and Δ*micA* were reproducible, results from separate immunolabelling experiments were combined. The average Pal concentration was calculated for each sample in independent experiments. The Pal concentration had to be normalized to 100 au µm^−3^ for the WT since different lamps and exposure times were used in the experiments. The values found for the deletion strains were adjusted accordingly (see Table S2). The results are summarized in Fig. 5b. The deletion of Δ*prc* resulted consistently in a decrease in Pal concentration, albeit above the fluorescent signal detected in Δ*pal*. In Δ*micA*, the amount of Pal was reproducibly higher than in WT cells. However, the extent of this increase varied. We were unable to confirm our findings by western blot, as the Δ*micA* strain was liable to lysis during centrifugation. Therefore, we could not obtain a quantifiable number of cells in the pellet. Pelleting of cells used in immunolabelling experiments was successful as they were fixed.

Although Prc did not directly digest Pal, the absence of this protease from cells resulted in a decrease in Pal levels detected with immunolabelling. Hence, it appeared that Prc regulates the activity of another protein that specifically degrades or downregulates Pal abundance. Since a *prc* deletion resulted in a stable Pal concentration of up to 65% of the cell cycle division age and the fluctuations in Pal concentration in WT cells were substantially diminished, this target of Prc may have the cell cycle phase-specific activity that we are looking for.

Based on the results from immunolabelling experiments, it can be concluded that the removal of sRNA MicA leads to an upregulation of Pal expression. However, the variation in Pal concentration during the cell cycle is not lost in Δ*micA*, making it unlikely that MicA has a role in the temporal regulation of Pal abundances during the cell cycle.

## Discussion

In the current work, the spatiotemporal behaviour of innate Pal during the cell cycle was studied by immunolabelling [40,41, 44]. This technique is complementary to the nowadays more commonly used fluorescent protein fusions, with the same type of readout: localized fluorescence signal in cells that can be detected with fluorescence microscopy. Both methods come with trade-offs that can affect the results. Any fluorescent protein fusion can suffer from steric hindrance caused by bulky fluorescent proteins (~25–30 kDa), this can affect potential interactions of the native protein with partner molecules. Fused fluorescent proteins can additionally change the expression levels or protein abundance. The main downside of immunolabelling is that it requires the fixation and permeabilization of cells. It does, however, allow the detection of endogenous proteins at endogenous levels and is therefore complementary to studies performed with fluorescent protein fusions [35]. The accumulation of TolB and Pal at the division site was confirmed in our study, and Pal retention at the new cell pole was detected to a greater extent than previously with fluorescent fusions [24,26]. These findings are not novel but reinforce that the Pal fluorescent protein fusions used in the work of others in our field were indeed behaving as endogenous proteins. The cell division machinery is assembled in two time-separated steps [45,46]. First, FtsZ and its membrane-attaching partners ZipA and FtsA, with several Z-ring-associated proteins, form the so-called proto-ring, which localizes at midcell [47]. After ~20% of the cell division cycle of cells grown in a minimal glucose medium, the proto-ring is followed by all other proteins involved in septum synthesis. To determine whether Pal belongs to the early or late divisome localizing proteins, we analysed the timing of its midcell localization. In Fig. 2b, the demographics of FtsZ (the first protein to localize at midcell) and FtsN (the last essential component of the divisome to appear at midcell) are shown side-by-side with those of Pal and TolB. Pal localization follows the timing of the late localizing divisome proteins. It shows a slightly later localization than FtsN (Fig. 2b). It does not show the dissociation shortly before the actual division, as seen for FtsN. Pal remains attached to the newly formed cell pole, which is not seen for other proteins. TolB accumulation appears to commence early in the cell cycle.

The retention of Pal at the new cell pole, as seen in our demographics from immunolabelling, persists longer than in demographics from earlier work with Pal-mCherry [26]. However, the time-lapse data of Pal-mCherry shown in [26] also show that the retention of Pal at the new cell pole lasts until the accumulation in the new division site. The retention of Pal at the newly formed cell poles further substantiates the model where the accumulated Pal at the division site is static because it is attached to PG, and it needs TolB to be detached and become mobile again [11,15]. As seen in Table S1, there is one TolB protein for every eight to ten Pal proteins [20]. Thus, it is plausible that the Pal proteins are detached from the new cell pole only after cell division has been completed. It ensures prolonged stabilization of the cell envelope at the new cell pole.

During the preparation of this manuscript, Hale *et al.* showed that GFP-TolA and TolQ-GFP require the synthesis of septal PG for their accumulation at midcell. TolA and TolQ fusions no longer localized to the middle of cells after two mass doublings of treatment with the same concentration of aztreonam (1 µg ml^−1^) used in this study [28]. It can therefore be assumed that Pal, which is dependent on TolA and TolQ for its localization, no longer accumulates at the septum of cells treated with aztreonam because of their absence [11,27, 28] rather than it being a direct effect of FtsI inhibition. This again is a replication of earlier work, with a complementary method, proving that endogenous Pal also requires active FtsI for its recruitment to the middle of the cell.

The cellular concentration of Pal changed during the cell division cycle. It decreased in the first stages of the cell cycle, but it increased again in the second half of the cell division cycle (Fig. 3). This finding is novel and adds another dimension to the spatiotemporal behaviour of Pal: not only the location of Pal but also its abundance changes during the cell cycle. This regulation of the abundance during the cell cycle has also been described for FtsZ, PBP3 and PBP5 [35]. The search for a regulator of the abundance of Pal during the cell cycle focussed on the periplasmic protease Prc and the sRNA MicA. The deletion of *micA* resulted in an overall increase of Pal in the cell, detected with immunolabelling, which supports the predicted role of MicA as a negative regulator of Pal [48,49]. However, this was a global effect, and cells maintained the initial decrease of Pal abundance and increase in later cell division stages. Deletion of *prc* did affect the change in abundance of Pal detected with immunolabelling during the cell cycle. The decline in Pal concentration in the early stages of the cell cycle was lost (Fig. 5a), the overall concentration of Pal was decreased and the increase during division was substantially reduced (Fig. 5b). Both MicA and Prc should be complemented in their respective deletion strains to confirm that this restores Pal abundance. The protease Prc is likely not a direct regulator of Pal because the detected amount of Pal decreased and did not increase as a consequence of the deletion of *prc* deletion. NlpI and Prc also control the abundance of MltD and MepS [50]. MltD is a lytic transglycosylase, and MepS is an endopeptidase; these two hydrolases are implied in the opening up of PG to ensure new material can be inserted and cells can grow [50]. NlpI and Prc can proteolyse MltD and MepS to ensure that the opening of PG only occurs when this is necessary for growth [50], and the authors speculate that the level of MltD and MepS degradation might also change during the cell cycle. Prc could adopt a similar role in regulating the regulator of Pal. Prc could indirectly keep the Pal levels early in the cell cycle low, but allow them to increase as the septum is formed and the OM needs to be stabilized to the PG.

The work presented here consolidates earlier work investigating the spatiotemporal behaviour of Pal relying on fluorescent protein fusions but also presents a new dimension of this behaviour: the abundance of Pal changes during the cell cycle. We have probed two putative regulators of this change in abundance: the sRNA MicA and the periplasmic protease Prc. We did not uncover the regulator of the change in abundance but hope our work can be a starting point for others to find out which factor(s) regulated Pal during the cell cycle.

## supplementary material

10.1099/acmi.0.000759.v3 Uncited Supplementary Material 1.

## Abbreviations

IM: inner membrane
NCBI: National Center for Biotechnology Information
OM: outer membrane
Pal: peptidoglycan-associated lipoprotein
PBP3: penicillin-binding protein 3
PG: peptidoglycan
PMF: proton motive force
sRNA: small RNA
