# Supplementary material for: Cell division cycle fluctuation of Pal concentration in Escherichia coli
Source: Access Microbiol. 2024 Nov 13;6(11):000759.v3. doi: 10.1099/acmi.0.000759.v3 (PMC11559426; doi:10.1099/acmi.0.000759.v3)
Supplement: Uncited Supplementary Material 1. [file acmi-6-00759-s001.pdf]

Supplementary material

Cell division cycle fluctuation of Pal concentration in *Escherichia coli*

Laureen M.Y. Mertens[1], Xinwei Liu[1], Jolanda Verheul[1], Alexander Egan[2], Waldemar Vollmer[2,3], Tanneke den Blaauwen[1*]

[1] Bacterial Cell Biology and Physiology, Swammerdam Institute for Life Science, University of Amsterdam, Science Park 904, 1098 XH Amsterdam, The Netherlands

[2] Centre for Bacterial Cell Biology, Biosciences Institute, Newcastle University, Newcastle upon Tyne, UK.

[3] Institute for Molecular Bioscience, The University of Queensland, Brisbane, QLD, Australia.

[*]Corresponding author: t.denblaauwen@kpnmail.nl

Table S1. Number of proteins produced per cell cycle from the two *tol-pal* operons and other OM-PG linking proteins, determined from ribosome profiling data (1). MOPS Complete is a rich medium, and MOPS Minimal is a glucose poor medium. The exact composition and other growth conditions can be found in the reference.

	**Medium**	
**Protein**	**MOPS Complete**	**MOPS Minimal**
YbgC	790	244
TolA	476	133
TolB	6,049	2,491
TolR	1,667	392
TolQ	864	221
Pal	64,020	17,960
CpoB	5,262	1,511
Lpp	1,191,641	619,492
OmpA	207,618	69,396

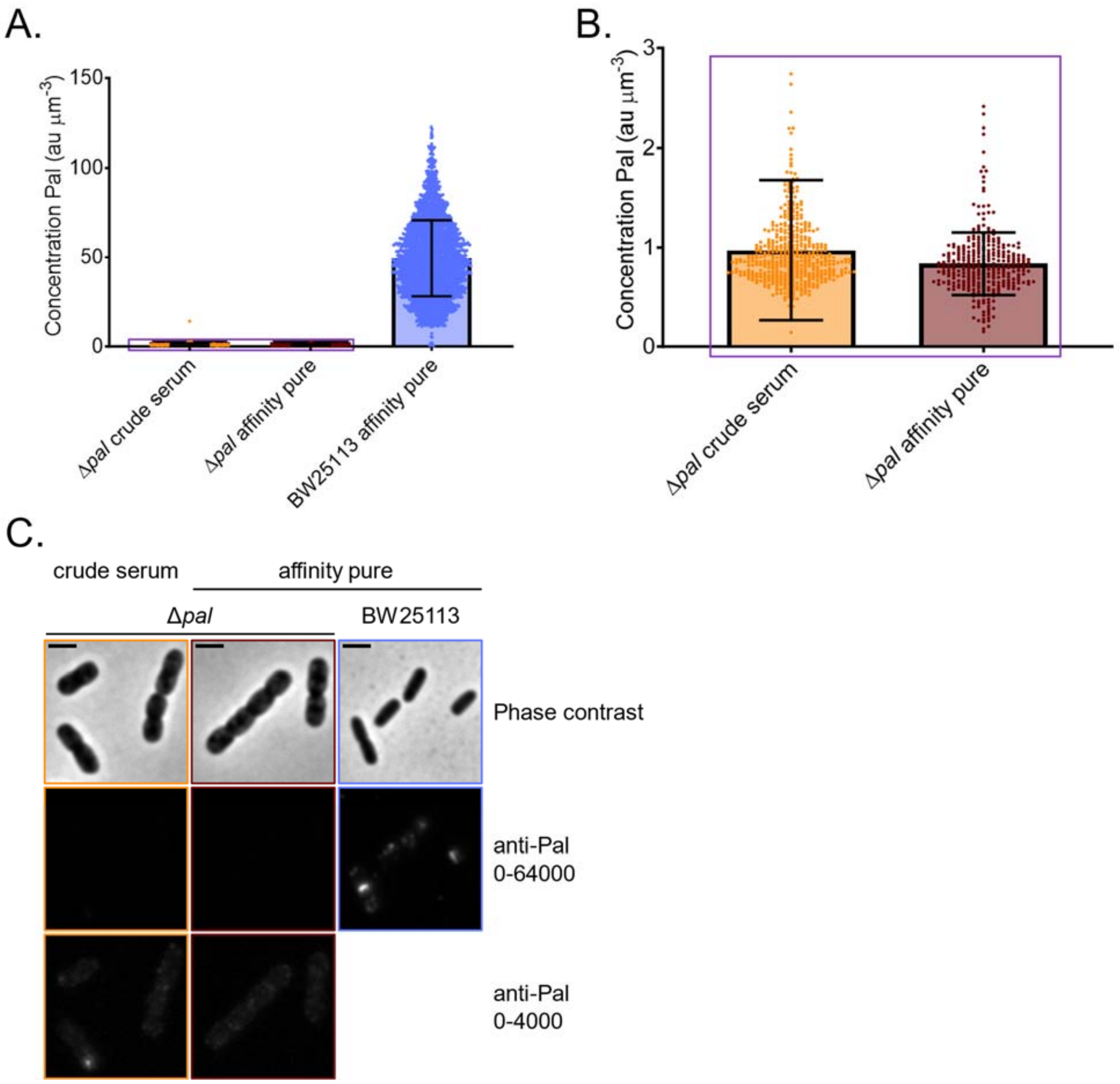

Fig S1. Anti-Pal can specifically recognise Pal in an immunolabeling assay.
Antibodies (crude serum) were adsorbed to a strain lacking the *pal* gene (Δ*pal*) grown in TY medium at 37°C (2). The antibodies that did not bind to these cells – present in the supernatant (affinity purified) – were used for immunolabeling its wildtype *E. coli* strain (BW25113) grown to steady state in minimal glucose medium (GB1) at 28 °C and for labelling an unlabelled batch of Δ*pal*. **A.** Bar graph of the Pal concentration (in au.µm^{-3}) in individual cells (shown as round markers) as well as the mean (horizontal line) and standard deviation (error bars) of each sample. N = 451, N = 320 and N = 3032 for the samples Δ*pal* crude serum, Δ*pal* affinity pure and BW25113 affinity pure, respectively. **B.** Figure A, but with an adapted Y-axis range, so one could observe differences between the Δ*pal* samples. **C.** Representative microscopy images. Brightness and contrast of the fluorescence channel were normalised per row (the lowest row has 16-fold enhanced fluorescence). The scale bar represents 2 µm.

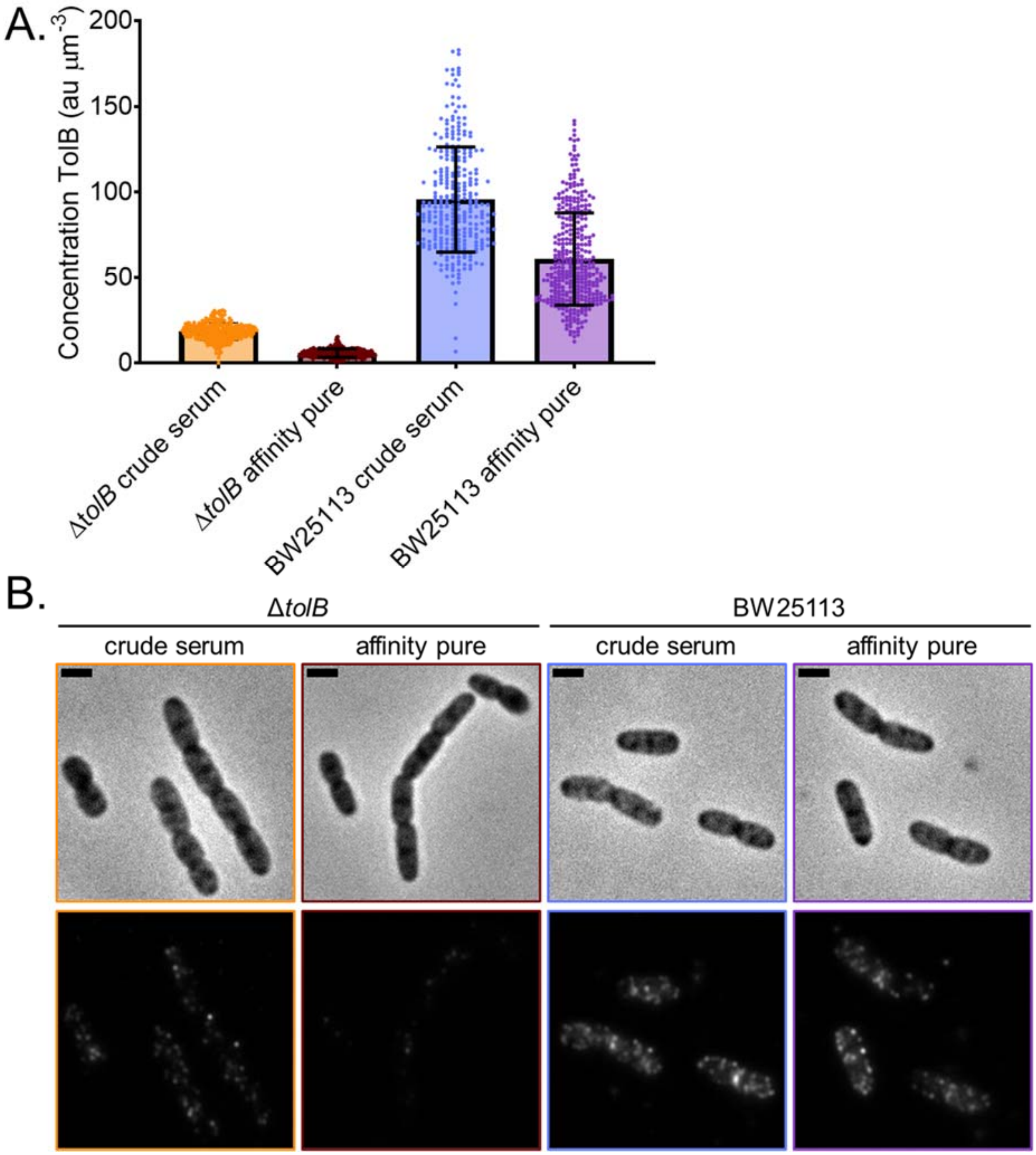

Fig S2. Anti-TolB can specifically recognise TolB after affinity purification with a Δ*tolB* strain.
Antibodies (crude serum) were adsorbed to a strain lacking the *tolB* gene (Δ*tolB*) and its wild-type strain BW25113 grown in TY medium at 37°C (2). The antibodies that did not bind to the Δ*tolB* cells – present in the supernatant (affinity purified) – were then used for immunolabeling BW25113 and Δ*tolB*. (A) Bar graph of the TolB concentration (in au.μm^{-3}) in individual cells (shown as round markers) as well as the mean (horizontal line) and standard deviation (error bars) of each sample. N = 271, N = 272, N = 303, and N = 375 for the samples Δ*tolB* crude serum, Δ*tolB* affinity pure, BW25113 crude serum and BW25113 affinity pure, respectively. (B) Representative microscopy images with normalised brightness and contrast for the fluorescence channel. The scale bar represents 2 µm.

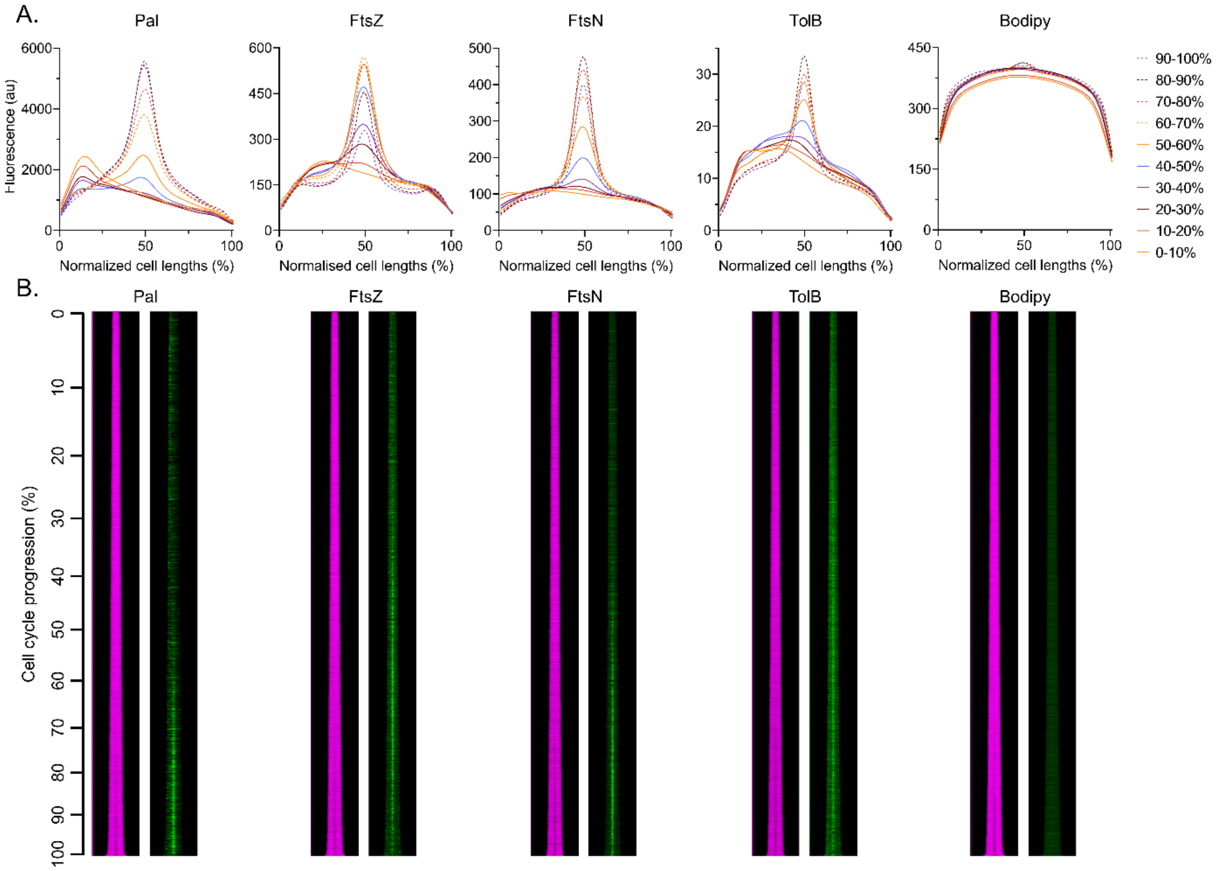

Fig S3. Fluorescence profiles and sorted demographs.

(A) Fluorescence profiles – as a function of normalised cell length – of cells labelled with antibodies without shifting up the profile of each age group as in main text Fig 2A and 2D. Cells were labelled with specific antibodies against (from left to right) Pal, FtsZ, FtsN and TolB and the membrane stain Bodipy. Cells were binned in 10 % age groups, and for each group, an average fluorescence profile was made and plotted against the normalised cell length. (B) Sorted fluorescence demographs (green) alongside their corresponding diameter demographs (in magenta), which were omitted in the main text Fig 2C. Cells were sorted based on cell length (shortest cells at the top, longest at the bottom; cell cycle progression indicated on the left), and their most fluorescent half is oriented to the left.

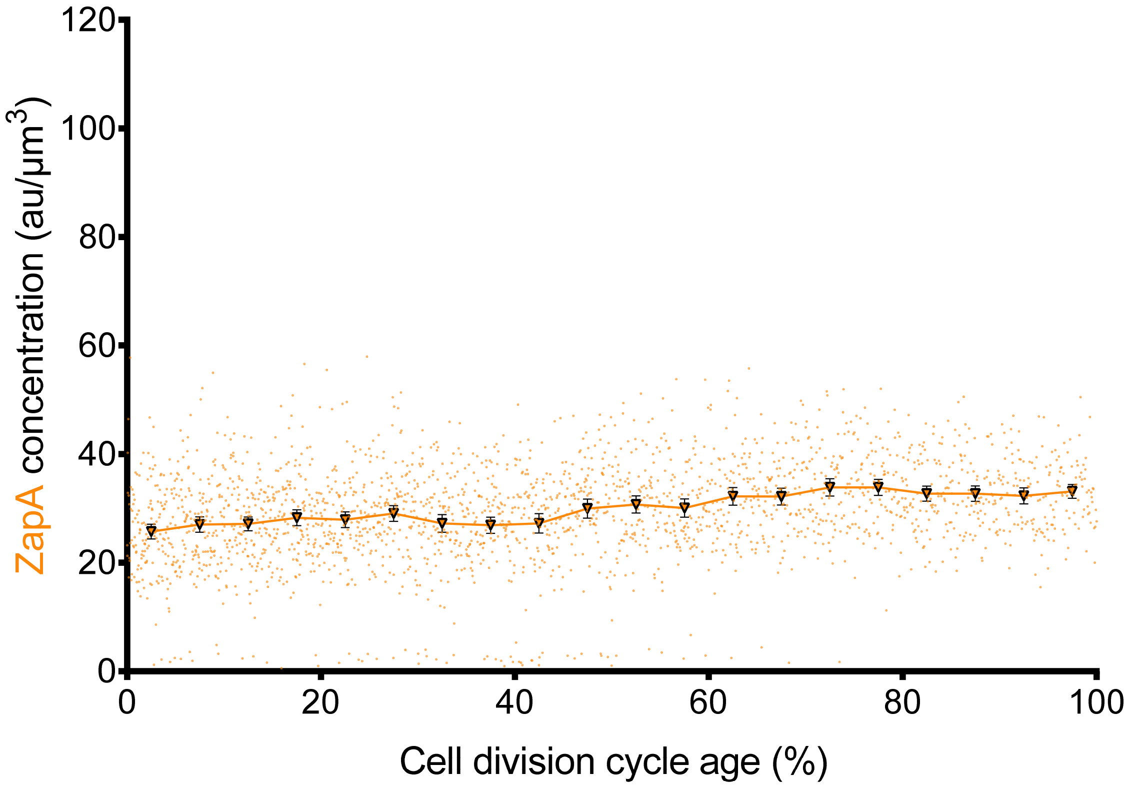

Fig S4. All cells labelled with anti-ZapA.

The graph includes 63 cells labelled with anti-ZapA that had a concentration <8.0 au μm^{-3}, omitted in the main text Fig 3.

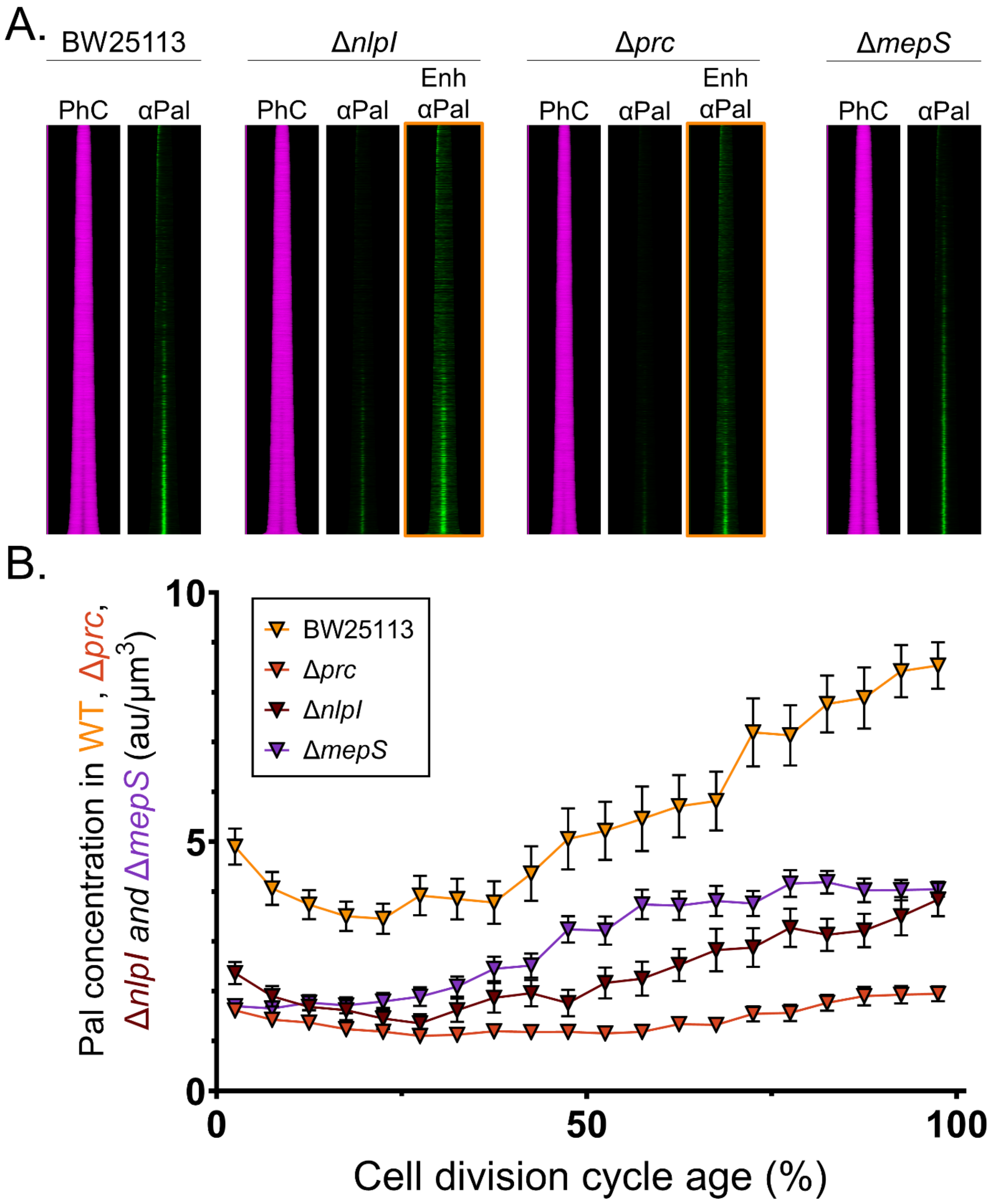

Fig S5. In the absence of MepS, NlpI and Prc, Pal-concentration in cells decreases.
(A) According to cell length sorted demographs of Pal-immunolabeled WT strain BW25113 alongside those of deletion stains Δ*nlpI,* Δ*prc*, and Δ*mepS*. Magenta demographs show the diameter, while the green demographs show the fluorescence along the cell length – cells are oriented with their most fluorescent pole to the left. Since fluorescence levels of Δ*nlpI* and Δ*prc* were too low to see Pal localisation in the demographs, an extra demograph with enhanced fluorescence was added (orange outline). (B) The concentration of Pal in arbitrary units per μm^3 plotted against the normalised cell division cycle age. The connected triangular markers correspond to the mean

Pal concentration for 5% age bins, with their 95% confidence interval displayed as error bars. The colours of the markers correspond to BW25113 (bright orange, N = 3298 cells), Δ*nlpI* (muted orange, N = 3084), Δ*prc* (dark red, N = 3883) and Δ*mepS* (lilac, N = 3084). Representative microscopy images can be found in Fig S6.

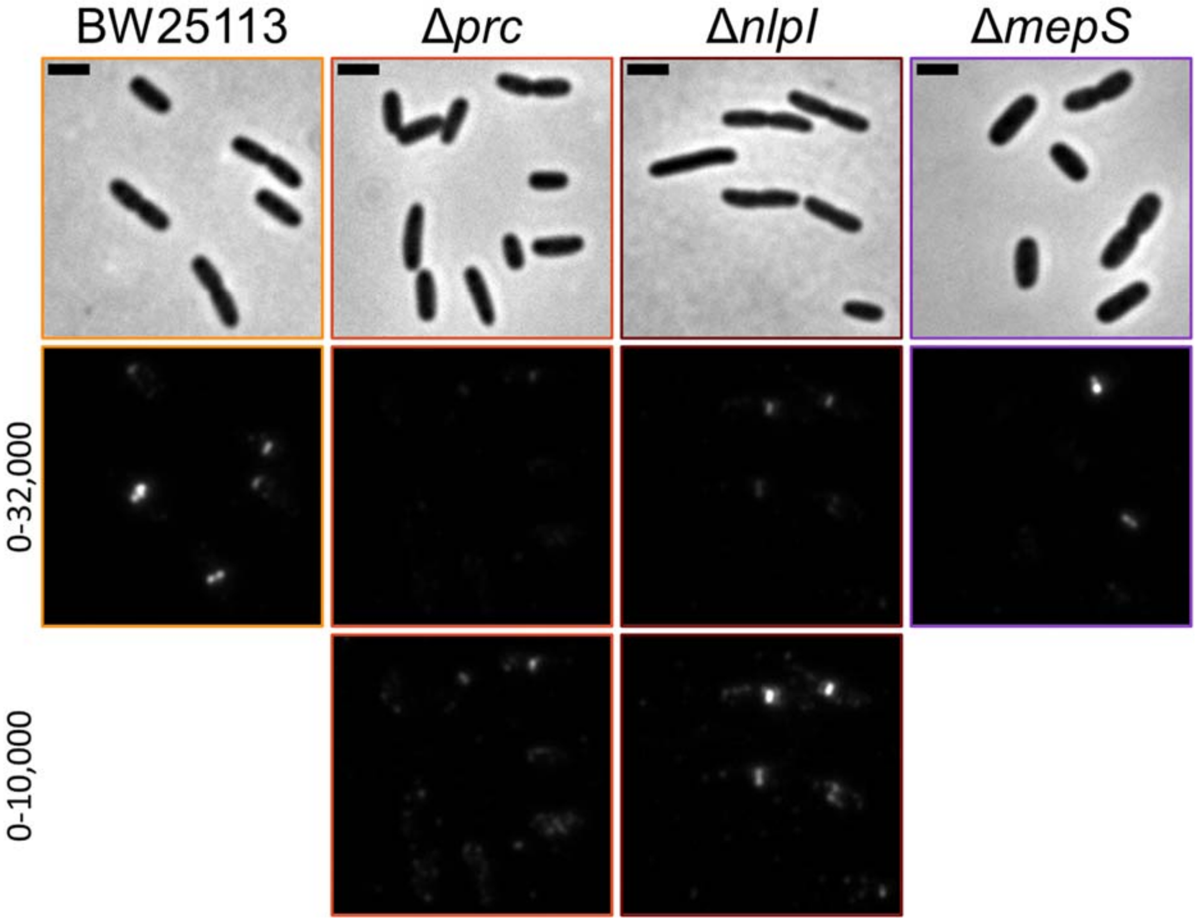

Figure S6. Representative microscopy images of BW25113, Δ*prc*, Δ*nlpI*, and Δ*mepS* labelled with anti-Pal (of the samples used in Fig S5). Cells were grown to steady state in GB-1 medium at 28°C and immunolabeled with αPal. Phase contrast images are shown on top; the corresponding fluorescence microscopy images is shown below. The middle row shows fluorescence images with normalised brightness and contrast for the fluorescence channel (0-32,000). The bottom row shows fluorescence images with enhanced brightness and contrast (0-10,000), so the localisation pattern of the less bright signal of αPal in Δ*prc* and Δ*nlpI* can be seen. The scale bars represent 2 μm.

Table S2. The concentration of Pal signal in WT (BW25113), Δ*pal*, Δ*prc* and Δ*micA* across several experimental sets included in main text Fig 5B.
Measured values were normalised by converting the mean concentration found for the WT sample to 100% (when there are multiple BW25113, the average mean was used). The factor calculated by dividing 100% by the measured mean of BW25113 was then used to convert the mean concentration (in au·µm^{-3}) to a percentage of the concentration of Pal in WT. Datasets 3 and 4 contained non-permeabilised cells (see Fig S7C and S7D), which were omitted for the final data calculation. For transparency, the complete data, including the nonpermeabilised cells are also shown (in grey). The omitted cell did not significantly affect the final result. The # indicates the datasets shown in the main text Fig 5A.

		Measured values for Pal concentration (au·µm^{-3})			Factor per set	WT mean = 100%	
		Mean	SD	N		Mean	N
**Set 1**					55.4785		
	WT#	1.80	1.15	3519		100	3519
	Δ*pal* 1#	0.31	0.09	3941		17.43	3941
	Δ*pal* 2	0.29	0.08	3610		16.31	3610
	Δ*prc* #	1.23	0.67	6752		68.74	6752
	Δ*micA* 1#	3.97	2.42	2217		220.28	2217
	Δ*micA* 2	4.56	2.59	2304		252.89	2304
**Set 2**					74.01103		
	WT 1	1.13	0.66	2407		83.88	2407
	WT 2	1.57	0.96	2463		116.12	2463
	Δ*prc* 1	0.82	0.42	2172		60.44	2172
	Δ*prc* 2	0.93	0.51	4013		68.86	4013
**Set 3**					34.36308		
All cells	WT	2.91	2.22	1530		100	1530
All cells	Δ*micA*	5.05	3.59	1237		173.59	1237
					29.59893		
>0.5	WT	3.39	2.07	1308		100	1308
>0.5	Δ*micA*	6.11	3.05	1017		180.91	1017
**Set 4**					25.06266		
All cells	WT	3.99	2.83	3495		100	3495
All cells	Δ*micA*	6.80	4.48	2532		170.406	2532
					22.57285		
>0.8	WT	4.43	2.67	3127		100	3127
>0.8	Δ*micA*	8.11	3.70	2110		183.16	2110

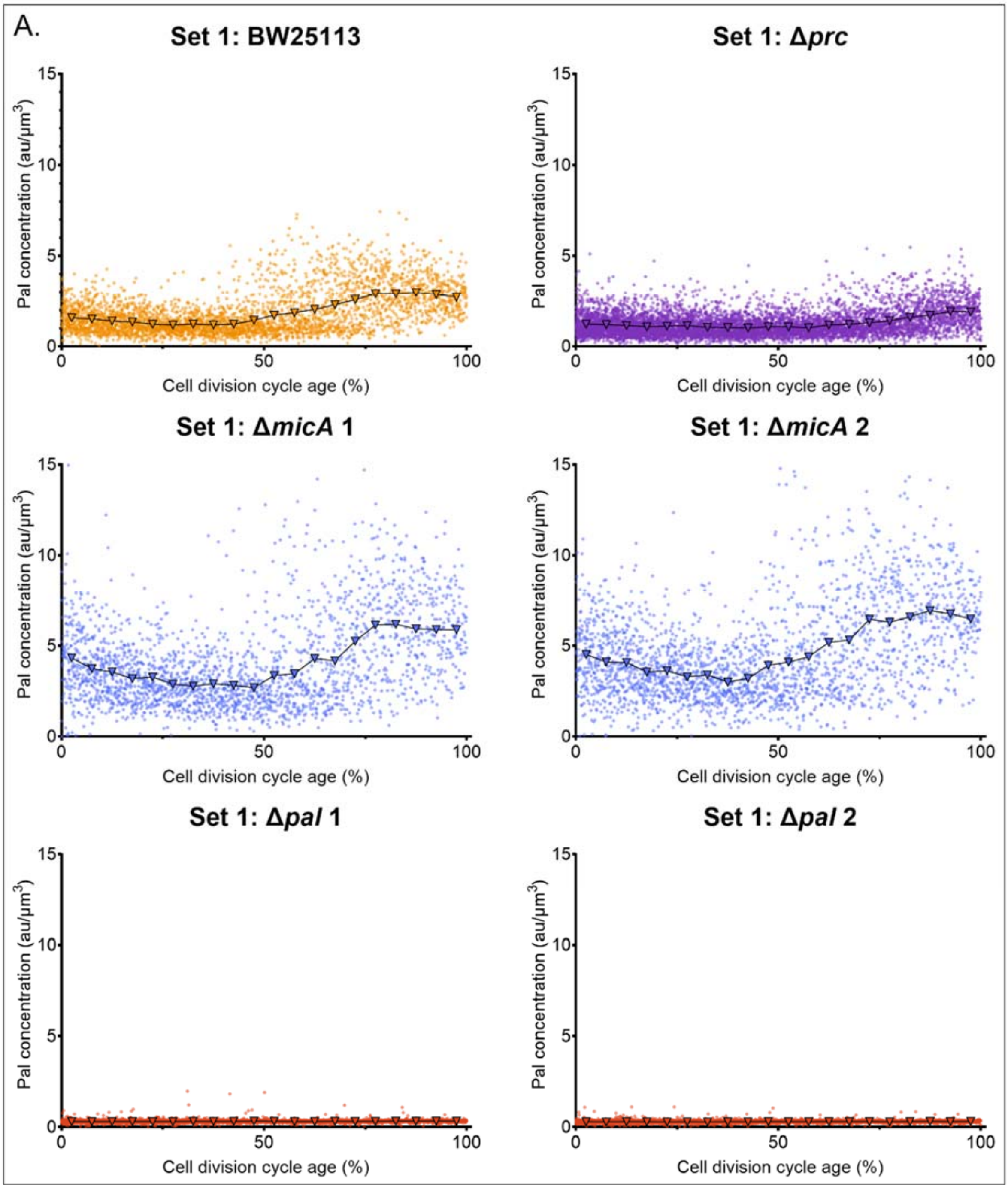
A.
Set 1: BW25113
Set 1: Δprc
Set 1: ΔmicA 1
Set 1: ΔmicA 2
Set 1: Δpal 1
Set 1: Δpal 2
Pal concentration (au/µm3)
Cell division cycle age (%)
0
5
10
15
0
50
100

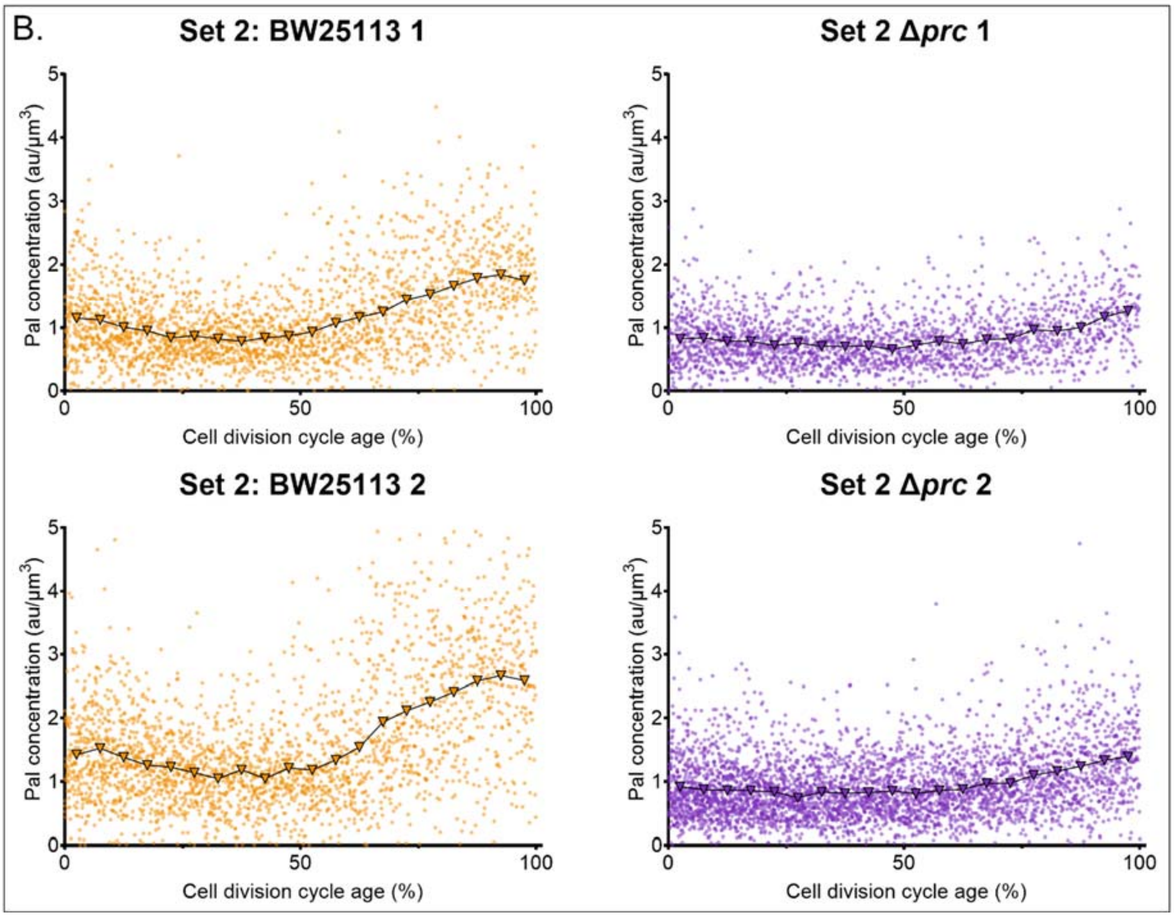
B.
Set 2: BW25113 1
Set 2 Δprc 1
Set 2: BW25113 2
Set 2 Δprc 2
Pal concentration (au/μm3)
Cell division cycle age (%)
0
1
2
3
4
5
50
100

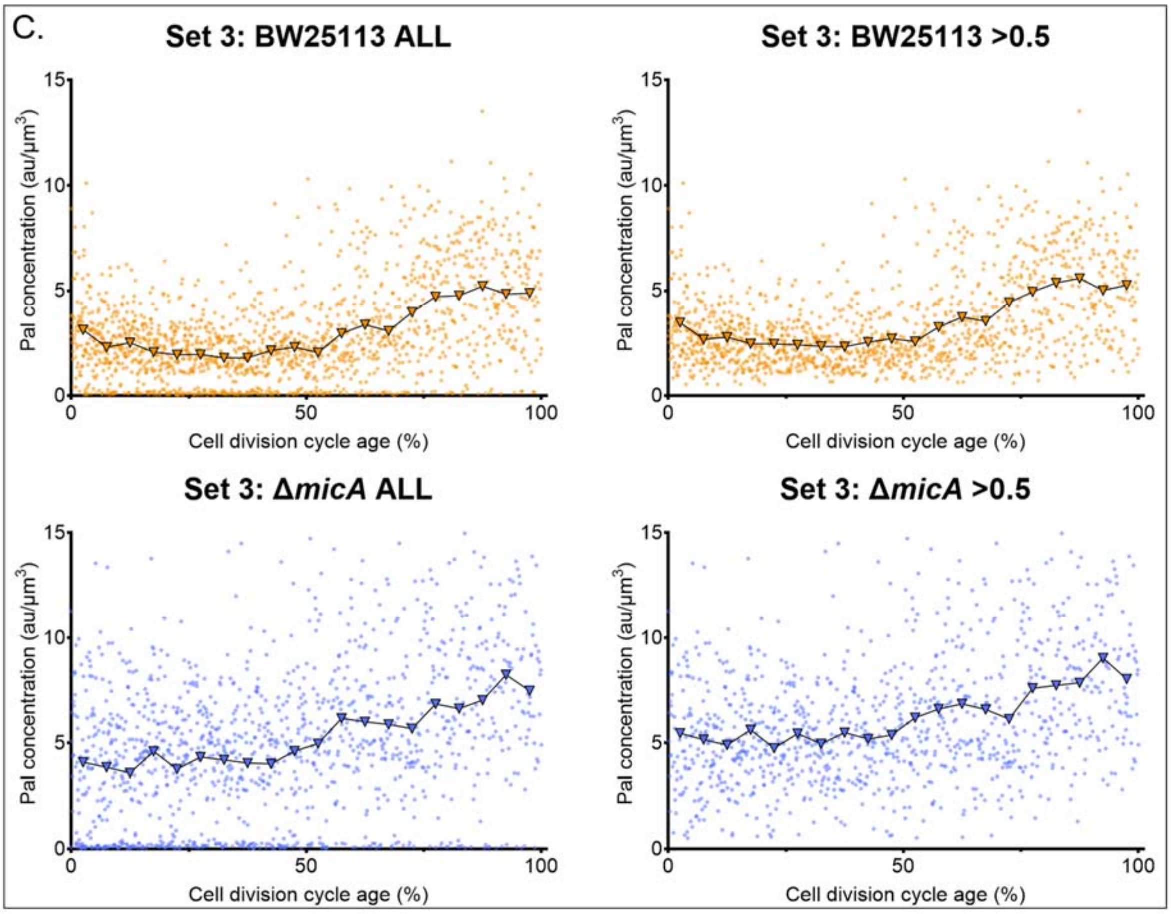
C.
Set 3: BW25113 ALL
Set 3: BW25113 >0.5
Set 3: ΔmicA ALL
Set 3: ΔmicA >0.5
Pal concentration (au/µm³)
Cell division cycle age (%)

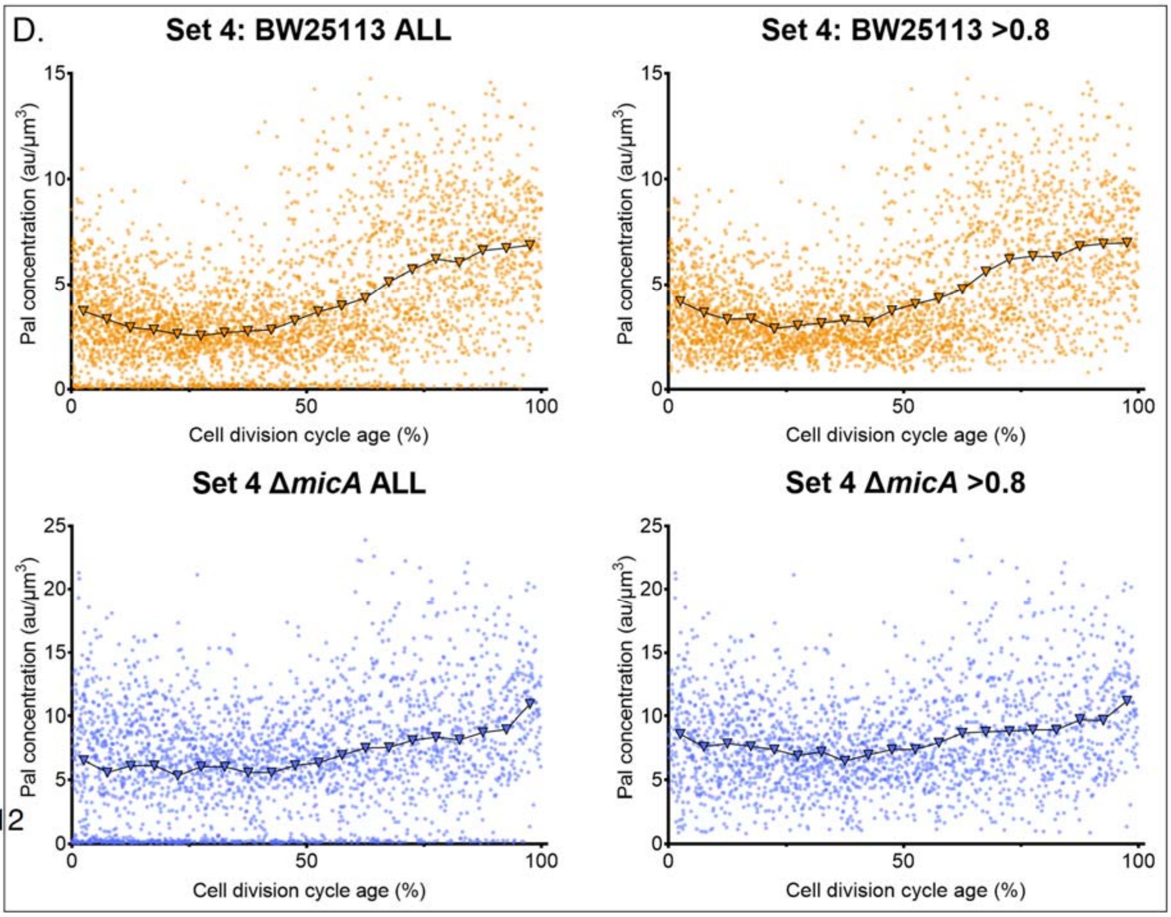
D.
Set 4: BW25113 ALL
Set 4: BW25113 >0.8
Set 4 ΔmicA ALL
Set 4 ΔmicA >0.8
Pal concentration (au/µm³)
Cell division cycle age (%)

Fig S7. Pal concentration in au.µm^{-3} per 5% age bin. Underlying experiments of main text Fig 5B.

Samples are described in Table S2 above. (C) and (D) All samples in both experiments contained a faction of unlabelled cells (see left panel for each sample), which were removed from the data (see right panel for each sample).

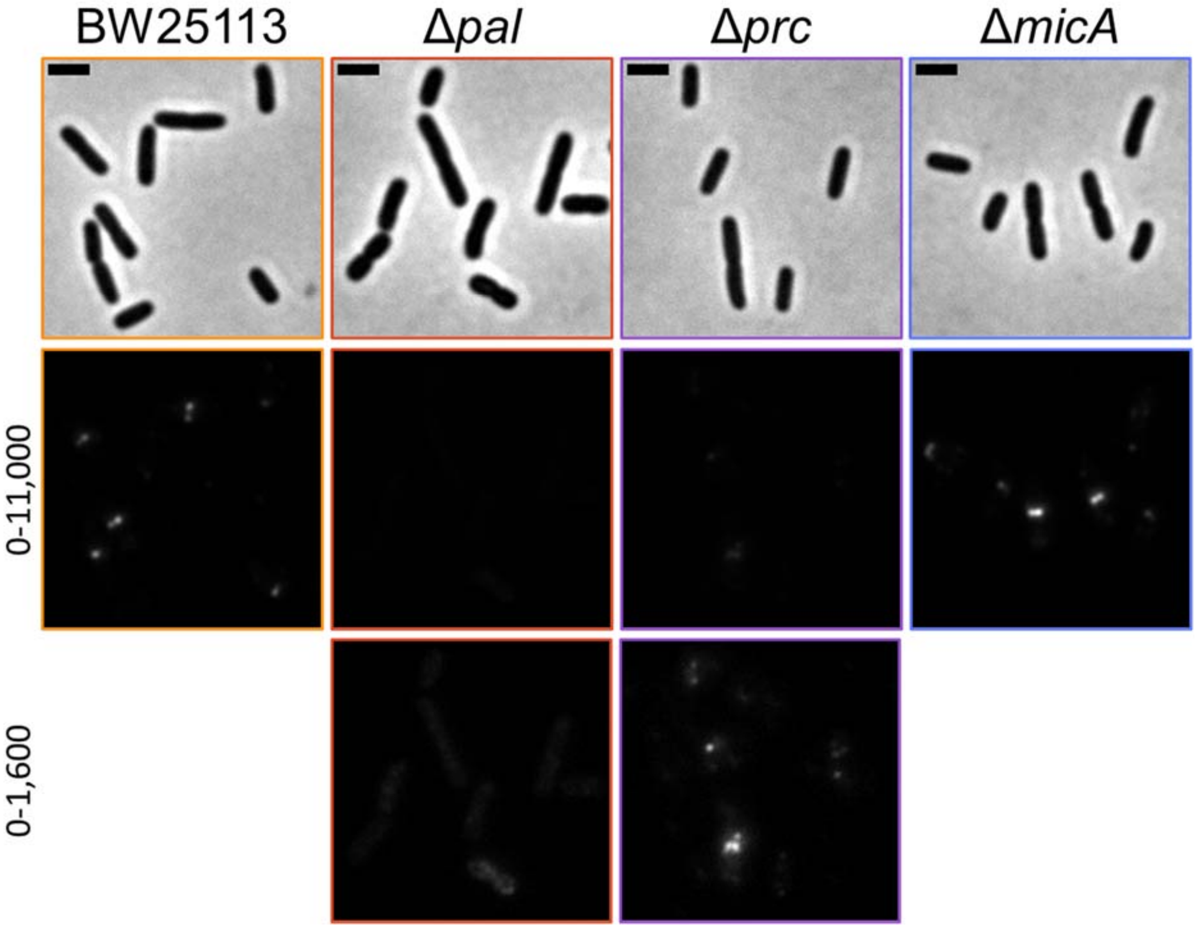

Fig S8. Representative microscopy images of BW25113, Δ*pal*, Δ*prc*, and Δ*micA* labelled with αPal (of the samples used in Fig 5A). Cells were grown to steady state in GB-1 medium at 28°C, immunolabeled with αPal. Phase contrast images are shown on top; the corresponding fluorescence microscopy images are shown below. The middle row shows fluorescence images with normalised brightness and contrast for the fluorescence channel (0-11,000). The lowest row shows the pictures with enhanced brightness and contrast (0-1,600), so the localisation pattern of the less bright signal of αPal in Δ*pal* and Δ*prc* can be seen. The scale bars represent 2 µm.

References

1. Li GW, Burkhardt D, Gross C, Weissman JS. Quantifying Absolute Protein Synthesis Rates Reveals Principles Underlying Allocation of Cellular Resources. Cell. 2014 Apr;157(3):624–35.

2. Baba T, Ara T, Hasegawa M, Takai Y, Okumura Y, Baba M, et al. Construction of *Escherichia coli* K-12 in-frame, single-gene knockout mutants: the Keio collection. Mol Syst Biol [Internet]. 2006 Jan [cited 2023 Apr 2];2(1). Available from: https://onlinelibrary.wiley.com/doi/10.1038/msb4100050
